# Nuclear Smad6 promotes gliomagenesis by negatively regulating PIAS3-mediated STAT3 inhibition

**DOI:** 10.1038/s41467-018-04936-9

**Published:** 2018-06-27

**Authors:** Jiantong Jiao, Rui Zhang, Zheng Li, Ying Yin, Xiangming Fang, Xiaopeng Ding, Ying Cai, Shudong Yang, Huijun Mu, Da Zong, Yuexin Chen, Yansong Zhang, Jian Zou, Junfei Shao, Zhaohui Huang

**Affiliations:** 1 Center of Clinical Research, Wuxi People’s Hospital of Nanjing Medical University, Wuxi, Jiangsu 214023 China; 2 Department of Neurosurgery, Wuxi People’s Hospital of Nanjing Medical University, Wuxi, Jiangsu 214023 China; 3 Wuxi Institute of Translational Medicine, Wuxi People’s Hospital of Nanjing Medical University, Wuxi, Jiangsu 214023 China; 4 Department of Radiology, Wuxi People’s Hospital of Nanjing Medical University, Wuxi, Jiangsu 214023 China; 5 Department of Pathology, Wuxi People’s Hospital of Nanjing Medical University, Wuxi, Jiangsu 214023 China; 6 Department of Neurosurgery, Nanjing Brain Hospital of Nanjing Medical University, Nanjing, Jiangsu 210029 China; 7 Wuxi Cancer Institute, Affiliated Hospital of Jiangnan University, Wuxi, Jiangsu 214062 China

## Abstract

To date, the molecular mechanism underlying constitutive signal transducer and activator of transcription 3 (STAT3) activation in gliomas is largely unclear. In this study, we report that Smad6 is overexpressed in nuclei of glioma cells, which correlates with poor patient survival and regulates STAT3 activity via negatively regulating the Protein Inhibitors of Activated STAT3 (PIAS3). Mechanically, Smad6 interacts directly with PIAS3, and this interaction is mediated through the Mad homology 2 (MH2) domain of Smad6 and the Ring domain of PIAS3. Smad6 recruits Smurf1 to facilitate PIAS3 ubiquitination and degradation, which also depends on the MH2 domain and the PY motif of Smad6. Consequently, Smad6 reduces PIAS3-mediated STAT3 inhibition and promotes glioma cell growth and stem-like cell initiation. Moreover, the Smad6 MH2 transducible protein restores PIAS3 expression and subsequently reduces gliomagenesis. Collectively, we conclude that nuclear-Smad6 enhances glioma development by inducing PIAS3 degradation and subsequent STAT3 activity upregulation.

## Abstract (web summary)

In glioma STAT3 signaling contributes to gliomagenesis. Here, the authors show that Smad6 expression correlates with poor survival and is overexpressed in glioma cells, and regulates STAT3 activity via negatively regulating PIAS3.

## Introduction

Glioma is the most common and fatal form of malignant brain tumor. Malignant gliomas are diffuse, highly invasive tumors with poor prognosis. For example, glioblastoma multiforme (GBM), grade IV of glioma, is the most aggressive and lethal glioma with a 5-year survival rate < 5%, despite complete surgical resection followed by radiation and chemotherapy^1^. The occurrence of gliomas is frequently associated with molecular changes involving epidermal growth factor receptor (EGFR) and phosphoinositol 3-kinase (PI3K)/Akt/mTOR pathways, as well as mutations of the phosphatase and tensin homolog, p53, DNA repair enzyme O6-methylguanine-DNA methyltransferase, and isocitrate dehydrogenase-1 and -2. Recent studies defined signal transducer and activator of transcription 3 (STAT3) as a potent regulator of gliomagenesis by inducing angiogenesis, host immunosuppression, tumor invasion, and anti-apoptosis^1^. Constitutively active STAT3 frequently occurs in human gliomas and has been implicated in glioma stemness maintenance, chemoresistance, and metastasis^2–7^. Thus, targeting suppression of constitutively activated STAT3 has emerged as a potential new treatment for gliomas^2,4,8–10^.

STAT3 activation through phosphorylation is induced by a variety of cytokines and growth factors. Upon activation, STAT3 forms homodimers or STAT3/STAT1 heterodimers, and undergoes nuclear translocation and binding to the sis-inducible element (SIE), a promoter sequence, thereby inducing gene transcription. In normal cells, the protein inhibitors of activated STAT (PIAS) family (PIAS1, PIAS3, PIASx, and PIASy) regulates STAT activity. PIAS1 and PIAS3 bind activated STAT1 and STAT3, and prevent their ability to bind DNA^11^. Several studies have addressed the expression or function of PIAS3 in disease states, indicating that PIAS3 can counteract the function of constitutively active STAT3^8,12–14^. In GBM, loss of PIAS3 protein (not messenger RNA) contributes to enhanced STAT3 transcriptional activity and subsequent cell proliferation^12^. Transducible peptide of PIAS3 efficiently inhibits STAT3 signaling and subsequently GBM cell migration, proliferation, and survival^8,12^. However, the molecular mechanisms underlying PIAS3 loss in GBM are not yet clear.

Intracellular Smad family proteins transduce extracellular signals from transforming growth factor-β (TGFβ) superfamily members to the cell nucleus where they activate downstream gene transcription. Smads, which form a trimer of two receptor-regulated Smads (R-Smads), such as Smad2 and Smad3, and the co-Smad, Smad4, act as transcription factors to regulate gene expression. Among the Smad family, there are two inhibitory Smads, Smad6 and Smad7, and Smad6 generally mediates bone morphogenetic protein (BMP) signals, whereas Smad7 mediates TGFβ signaling^15–17^. Previous studies have demonstrated the key role of Smad7 in tumorigenesis^18–20^, whereas little is known concerning the role of Smad6 in human cancers, including in the glioma^21^.

In the present study, we observed that Smad6 levels were increased in nuclei of glioma cell and associated with poor patient survival. Functional analysis showed that overexpression of nuclear-Smad6 promotes tumorigenesis. Further mechanical investigations demonstrated that Smad6 is a novel PIAS3-interacting protein that antagonizes PIAS3-mediated STAT3 transcriptional inhibition by accelerating PIAS3 ubiquitination and degradation. Moreover, Smad6 MH2 transducible protein restores PIAS3 expression via competitive inhibition of Smad6 and subsequently reduces proliferation and stemness of GBM cells.

## Results

### Smad6 is upregulated and associated with glioma pathology

**Fig. 1:**
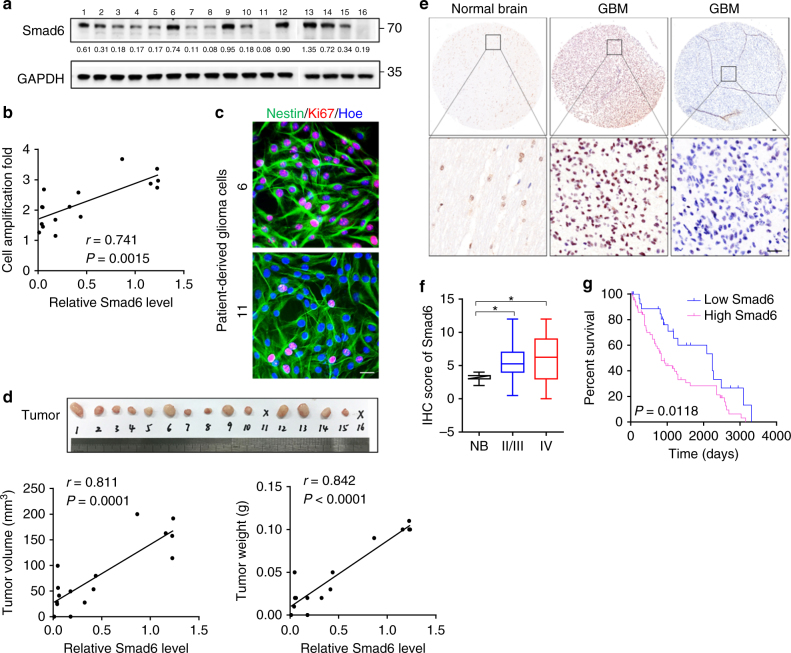
Smad6 is overexpressed in gliomas and predicts poor prognosis. **a** Smad6 expression in patient-derived glioma cells was examined by western blotting. GAPDH was used as a loading control. The relative quantification of Smad6 was listed. **b** Smad6 levels correlated to the proliferation capacity in patient-derived glioma cells (*P*- and *r*-values indicated, Spearman’s correlation analysis). Cells were cultured for 48 h and cell proliferation was monitored by a CCK-8 method. **c** Double immunofluorescence (IF) staining of Nestin and Ki67 in represented patient-derived glioma cells. Hoechst (Hoe) labeled the nuclei. Scale bars, 40 μm. **d** Smad6 expression correlated to tumor formation of patient-derived glioma cells (*P*- and *r*-values indicated, Spearman’s correlation analysis). Xenograft tumors derived from patient-derived glioma cells were shown in upper panel. The correlations of Smad6 to tumor volume and weight were shown in lower panel. **e** Immunohistochemistry (IHC) staining for Smad6 in glioma tissues. Scale bars, 50 μm. **f** Smad6 protein was frequently increased in nuclei of gliomas as compared it in normal brains (*P *<* 0.05, Unpaired *t*-test). **g** Overall survival analysis based on nuclear-Smad6 expression levels in glioma tissues. Groups were ranked according to nuclear-Smad6 IHC scores. The mean expression level of normal brains was used as a cutoff. The survival time of glioma patients with high Smad6 was significantly shorter than that of patients with low Smad6 (Log-rank *χ*^2^ = 6.348, *P* = 0.0118)

To determine the significance of Smad6 in human gliomas, we cultured primary cells derived from patient-derived gliomas tissue resections. Immunofluorescence (IF) showed that these patient-derived cells are Nestin/Glial fibrillary acidic protein (GFAP) double positive (Supplementary Figure 1a), confirming they origin from neurological tissues. Smad6 protein expression and proliferation ability were detected in these cells (Fig. 1a). Correlation analysis indicated that Smad6 protein levels positively correlated to the proliferative ability of these patient-derived glioma cells (Fig. 1b, c). To further investigate the contribution of Smad6 to glioma pathology, we established a patient-derived xenograft model. As shown in Fig. 1d, the expression levels of Smad6 in these cells were positively correlated to their tumorigenic potential and the xenografts with relatively higher levels of Smad6 grew more quickly than those with relatively lower Smad6. Then, we determined the subcellular expression of Smad6 through double IF staining. It showed that Smad6 is low expressed in astrocytes and mostly located in the nuclei (Supplementary Figure 1b). In primary glioma cells, Smad6 was also determined to be a predominantly nuclear protein and its expression intensity is corresponding to the tumor formation capacity (Supplementary Figure 1c). Then, we determined the cellular localization of Smad6 in normal human brain (Supplementary Figure 2a, b) and GBM tissues (Supplementary Figure 2c) by double IF staining. It indicated that Smad6 was a dominantly nuclear-expressing protein both in astrocytes and neurons in vivo. To further confirm the potential clinical implication of Smad6 in gliomas, immunohistochemistry (IHC) was performed using a glioma tissue microarray including 142 tumor samples and 7 normal samples. As shown in Fig. 1e, Smad6 was primarily localized in the nuclei of normal brain and glioma tissues. Thus, we proceeded with an analysis of nuclear-Smad6 expression in glioma tissues. It showed that Smad6 protein levels were substantially higher in glioma tissues from stage II/III to stage IV tissues than it in normal brains (Fig. 1f). The mean expression level of normal brains was used as a cutoff. Of the 142 tumors assessed, 81 (57.04%) presented higher Smad6 expression, 61 (42.96%) displayed lower Smad6 expression (Supplementary Data 1 and Supplementary Table 1). Importantly, survival analysis performed on 96 patients with detailed survival information indicated that higher Smad6 were closely associated with poorer clinical prognosis (Fig. 1h, Log-rank *χ*^2^ = 6.348, *P* = 0.0118) regardless of age and sex (Supplementary Data 1 and Supplementary Table 1). As the mean basic survival time is different between grade IV and lower grades of gliomas (Supplementary Figure 3a), the survival analyses were further performed separately based on two glioma groups as shown in Supplementary Figure 3b, c. Although no significance was found in grade II/III patients, the median survival time of patients with lower Smad6 (2483 days) is longer than the time of patients with higher Smad6 (1295 days). In grade IV gliomas, high Smad6 protein expression is closely associated with poor survival outcome. Therefore, our data suggest that the expression of Smad6 is associated with glioma tumorigenesis, especially in GBM.

### Smad6 is an oncoprotein in gliomas

**Fig. 2:**
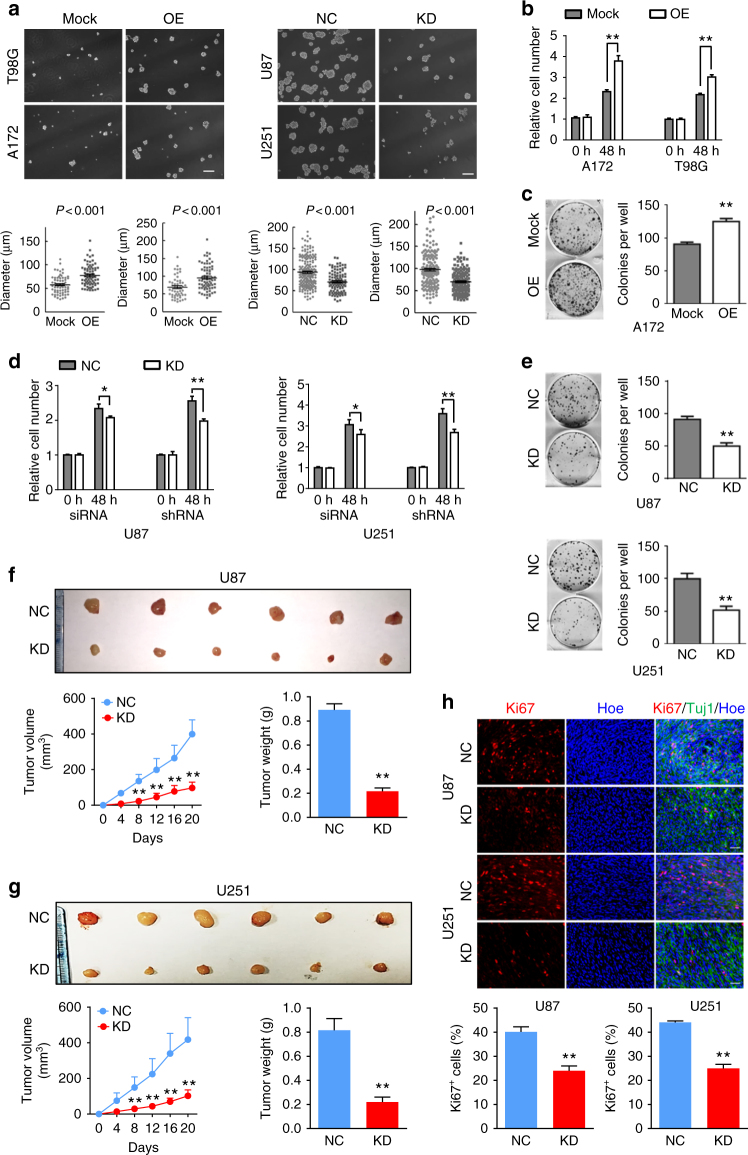
Smad6 promotes tumor sphere formation, cell proliferation and tumorigenesis of GBM cells. **a** Nuclear-Smad6 promoted tumor sphere formation of GBM cells. Representative images of in vitro tumor sphere formation in T98G and A172 cells with adenovirus mediated nuclear-Smad6 OE (upper panel) and U87 and U251 cells with lentivirus-mediated Smad6 knockdown (KD; lower panel). Scale bars, 200 μm. **b** Nuclear-Smad6 OE promoted cell growth in both A172 and T98G cells (*n* = 6). **c** Nuclear-Smad6 OE increased colony formation of A172 cells (*n* = 3). **d** Depletion of Smad6 by siRNA or Lenti-shRNA inhibited cell growth in both U87 and U251 cells. (*n* = 6). **e** Depletion of Smad6 impaired colony formation of U87 and U251 cells (*n* = 3). **f**,** g** Smad6 depletion inhibited tumor growth in nude mice. Mean tumor volumes and average tumor weight of xenografts of U87 (**f**) and U251 (**g**) with Smad6 KD were significantly decreased compared with their corresponding controls (*n* = 6). **h** Smad6 KD impaired proliferation of tumor cell derived from U87 and U251 cells. The representative double IF images of xenograft tumor sections with Ki67 and Tuj-1 (upper panel) and the quantification of (lower panel) the fraction of Ki67-positive cells. Positive cells were quantified *n* = 20 randomly selected fields per mouse (*n* = 6). Scale bars, 50 μm. Data were represented as means ± SD and analyzed using unpaired Student’s *t*-test in **a**, two-tailed Student’s *t*-test in **b**–**h**. **P < 0.01

To explore the functions of Smad6 in gliomas, established GBM cell lines were used in the following experiments. We first examined Smad6 expression in GBM cell lines using western blotting and IF staining. We observed that all four examined GBM cell lines expressed Smad6 (Supplementary Figure 5a). Meanwhile, IF staining indicated that Smad6 is predominately expressed in nuclei of U87 and U251 cells, and is distributed evenly from the cytoplasm to the nucleus of A172 and T98G cells (Supplementary Figure 5b). Based on the expression character of Smad6 in vivo and different cellular localization between the four GBM cell lines in vitro, these two sets of cell lines were appropriate cell model for Smad6 functional analysis. We generated Smad6 overexpression (OE) (Supplementary Figure 6a) and knockdown (KD) constructs (Supplementary Figure 6c). Adenovirus-delivered Smad6 (Flag-Smad6) was predominately expressed in the cytoplasm of T98G cells (Supplementary Figure 6b). Thus, to obtain a nuclear-forced Smad6 construct (Smad6-NLS), a tandem repeat of the nuclear localization signal (NLS) was fused at its N terminus to increase the translocation of Smad6 into the nucleus (Supplementary Figure 6, a, b). A172 and T98G were used to establish Smad6-NLS cell lines, and U87 and U251 cells were used to establish Smad6-depleted cell lines. As shown in Fig. 2a, forced expression of nuclear-Smad6 (Smad6-NLS, OE) significantly increased tumor sphere initiation and growth in A172 and T98G cells, whereas Smad6 KD significantly inhibited sphere formation in U87 and U251 cells. The promotive effects of Smad6 were also observed in secondary sphere formation (Supplementary Figure 7). Next, we observed that nuclear-Smad6 significantly enhanced A172 and T98G cell proliferation (Fig. 2b), accompanied with increased A172 colonies in semisolid medium (Fig. 2c). As a result of failed colony formation in soft agar, we did not evaluate the effect of nuclear-Smad6 OE on colony formation of T98G cells. Accordingly, Smad6 KD significantly suppressed cell growth and colony formation in U87 and U251 cells (Fig. 2d, e). To further verify the in vivo tumorigenesis-promoting effect of nuclear-Smad6, a xenograft tumor growth assay was performed in nude mice. Tumors derived from U87 and U251 cells with depleted Smad6 grew more slowly than those derived from the control cells (Fig. 2f, g). Ki67 staining further confirmed decreased proliferation in tumors with Smad6 KD (Fig. 2h). Taken together, these data indicate that nuclear-Smad6 promotes glioma cell growth and development.

### Smad6 regulates STAT3 activation through PIAS3 inhibition

**Fig. 3:**
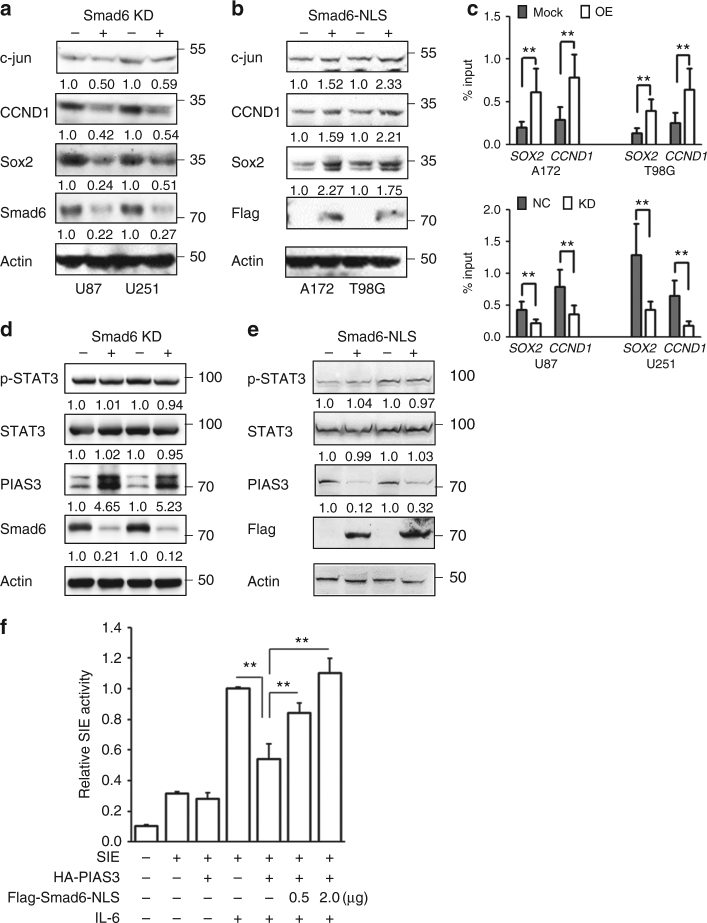
Smad6 promotes STAT3 activation through inhibition of PIAS3. **a**,** b** Nuclear-Smad6 regulated STAT3 downstream genes expression in GBM cells. The expression of proteins downstream of STAT3 signaling (c-jun, CCND1, and Sox2) were analyzed by western blot in glioma cells with Smad6 KD (**a**) or nuclear-Smad6 OE (**b**). The quantification of indicated proteins was listed. **c** Nuclear-Smad6 enhanced the enrichment of STAT3 on the promoter of STAT3 downstream targets. The enrichment of STAT3 binding to the promoter of target genes (*SOX2* and *CCND1*) was examined by ChIP-qPCR in glioma cells with nuclear-Smad6 OE (left panel) or KD (right panel) (*n* = 3). **d** Smad6 KD or **e** nuclear-Smad6 OE did not influence the phosphorylation (Y705) of STAT3, but regulated PIAS3 expression. **f** Nuclear-Smad6 rescued STAT3 transcriptional activity that was inhibited by PIAS3 (*n* = 3). Luciferase assay of STAT3 transcriptional activity in HEK293 cells transfected with HA-PIAS3 and/or indicated doses (μg) of Flag-Smad6-NLS. IL-6 (25 ng ml^−1^) was added 6 h before the assay. Data were represented as means ± SD and analyzed using two-tailed Student’s *t*-test. **P < 0.01

Constitutive STAT3 activation has been identified in GBM and is essential for maintenance of GBM stemness^2–7^. To examine whether Smad6 contributes to STAT3 activation, we first analyzed proteins downstream of STAT3 associated with glioma stemness, including c-jun, CCND1, and Sox2. The results showed that Smad6 depletion resulted in decreased expression of these proteins in U87 and U251 cells (Fig. 3a), whereas nuclear-Smad6 OE instigated upregulation of these proteins in A172 and T98G cells (Fig. 3b). We then determined whether nuclear-Smad6 promotes STAT3 transcriptional activity by enhancement of STAT3 DNA binding using a chromatin immunoprecipitation (ChIP) assay. As shown in Fig. 3c, nuclear-Smad6 OE increased the DNA binding of STAT3 to the promoters of *SOX2* and *CCND1* in A172 and T98G cells, whereas Smad6 KD decreased STAT3 DNA-binding ability to these promoters in U87 and U251 cells. As STAT3-dependent transcription was regulated by tyrosine phosphorylation of STAT3^22^, we determined whether Smad6 regulates STAT3 transcriptional activity through affecting its phosphorylation. Surprisingly, both phosphorylated STAT3 (Y705) and total STAT3 levels were not altered in GBM cells after Smad6 KD (Fig. 3d) or nuclear-Smad6 OE (Fig. 3e), suggesting a novel regulation mechanism of Smad6 on STAT3 activation in GBM. We observed that PIAS3, a natural inhibitor of STAT3, was negatively regulated by Smad6 (Fig. 3d, e). Smad6 depletion resulted in increased levels of PIAS3 in U87 and U251 cells (Fig. 3d). Conversely, Smad6-NLS OE resulted in decreased expression of PIAS3 in A172 and T98G cells (Fig. 3e). Previous studies revealed that PIAS3 functions as an endogenous STAT3 inhibitor through impairing its DNA-binding ability and subsequent transcription activation^23,24^. To investigate whether Smad6 enhances STAT3 activity via PIAS3 inhibition, we used a commercial STAT3 reporter system to monitor STAT3 activity in 293T cells. In this system, a luciferase reporter is driven by SIE, a promoter target sequence for STAT3 homodimers or STAT3/STAT1 heterodimers. As expected, we observed that interleukin (IL)-6-dependent transcription from SIE was inhibited by PIAS3 OE (Fig. 3f). Introduction of nuclear-Smad6 rescued IL-6-dependent transcription that was inhibited by PIAS3 in a dose-dependent manner. To investigate the effects of PIAS3 on GBM cell growth, we established PIAS3 KD or OE cell constructs using four GBM cell lines (Supplementary Figure 8a) based on their PIAS3 expression status (Supplementary Figure 5c, d). SIE reporter assays showed that PIAS3 served as a potent inhibitor for STAT3 by not only inhibiting constitutive STAT3 activity but also impairing EGFR-induced STAT3 transcription activation (Supplementary Figure 8b). Moreover, PIAS3 functioned to negatively regulate cell growth (Supplementary Figure 8c, d) and tumor sphere formation (Supplementary Figure 8e, f) in GBM cells, indicating that PIAS3 loss in GBM contributes to enhanced STAT3 activity and subsequent cell proliferation^12^. Taken together, our findings suggest that nuclear-Smad6 enhances malignant properties of human GBM cells through blocking PIAS3 inhibition of STAT3 activity.

### Smad6 negatively correlates to PIAS3 in gliomas

**Fig. 4:**
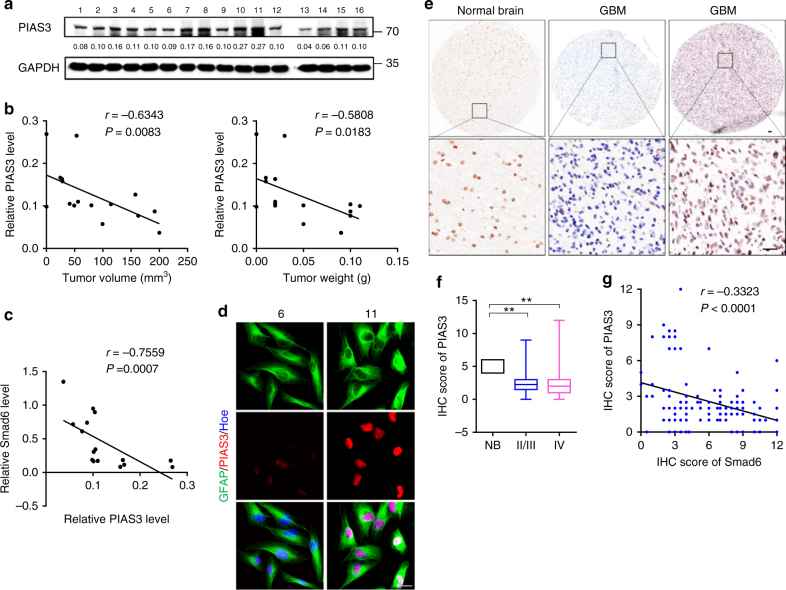
Smad6 negatively correlates to PIAS3 in gliomas. **a** PIAS3 expression in patient-derived glioma cells was examined by western blot. The relative quantification of PIAS3 was listed under the bands. **b** PIAS3 negatively correlated to tumor formation of patient-derived glioma cells (*P*- and *r*-values indicated, Spearman’s correlation analysis). **c** PIAS3 negatively correlated to Smad6 in patient-derived glioma cells (*P*- and *r*-values indicated, Spearman correlation analysis). **d** Double IF staining for PIAS3 and GFAP of patient-derived glioma cells. Hoechst (Hoe) labeled the nuclei. Scale bars, 20 μm. **e** IHC staining of for PIAS3 in glioma tissues. Scale bars, 50 μm. **f** PIAS3 protein was decreased in gliomas (**P *<* 0.01, Unpaired *t*-test). **g** PIAS3 expression negatively correlated with nuclear-Smad6 in primary glioma tissues (*P*- and *r*-values indicated, Spearman’s correlation analysis)

These observations promoted further assessments of the relationship between PIAS3 and Smad6 in human gliomas. First, we measured PIAS3 expression in patient-derived glioma cells (Fig. 4a) and revealed that PIAS3 expression was negatively correlated to the xenografts tumorigenic potential (Fig. 4b). Importantly, PIAS3 levels in these cells were negative correlated to Smad6 (Fig. 4c). Double IF staining of representative primary glioma cells which shown in Supplementary Figure 1c well documented a negative association between Smad6 and PIAS3 (Fig. 4d). PIAS3 was determined to be expressed in nuclei of primary glioma cells. We further determined the cellular localization of PIAS3. It indicated that PIAS3 is expressed in nuclei of normal astrocytes in vitro (Supplementary Figure 1b) and in vivo (Supplementary Figure 4a), cytoplasm of neurons (Supplementary Figure 4b) in vivo, and nuclei of glioma cells in GBM tissues (Supplementary Figure 4c). Moreover, the negative association between Smad6 and PIAS3 was also observed in GBM cell lines. A172 and T98G cells express relative higher PIAS3 compared to U87 and U251 cells (Supplementary Figure 5c, d), corresponding to their non-tumorigenic characteristic (according to ATCC cell backgrounds). To uncover the clinical implication of PIAS3, we detected PIAS3 expression in human glioma tissues using IHC (Fig. 4e). In view of different subcellular expression of PIAS3 between neuron and astrocyte/glioma cell, we determined the PIAS3 expression in nuclei in glioma tissues. It discovered that PIAS3 protein levels were substantially lower in glioma tissues from stage II/III to stage IV tissues than it in normal brains. Of the 142 tumors, 118 (83.1%) showed decreased PIAS3, 24 (16.9%) displayed increased PIAS3 expression (Fig. 4f and Supplementary Table 2). Survival analysis indicated that low PIAS3 protein level in gliomas was associated unfavorable survival outcome (Supplementary Figure 9a–c). Although no significance was found in grade IV patients, the median survival time of patients with higher PIAS3 (2240 days) is longer than that of patients with lower PIAS3 (727 days). Survival analysis based on The Cancer Genome Atlas (TCGA) GBM database using Project Betastasis (http://www.betastasis.com) confirmed that decreased *PIAS3* mRNA expression in GBM was associated with poor survival (Log-rank *χ*^2^ = 4.288, *P* = 0.0384, Supplementary Figure 9d). To investigate the potential regulation of Smad6 on PIAS3, we analyzed the correlation between Smad6 and PIAS3 (Fig. 4g). It showed that PIAS3 expression inversely correlated with nuclear-Smad6 in glioma tissues (Spearman’s *r* = − 0.3323, *P* < 0.0001). Thus, these observations, as well as the results shown in Fig. 3d, e, suggest that nuclear-Smad6 negatively regulates PIAS3 at the transcriptional level.

### Smad6 decreases PIAS3 via ubiquitin-proteasome pathway

**Fig. 5:**
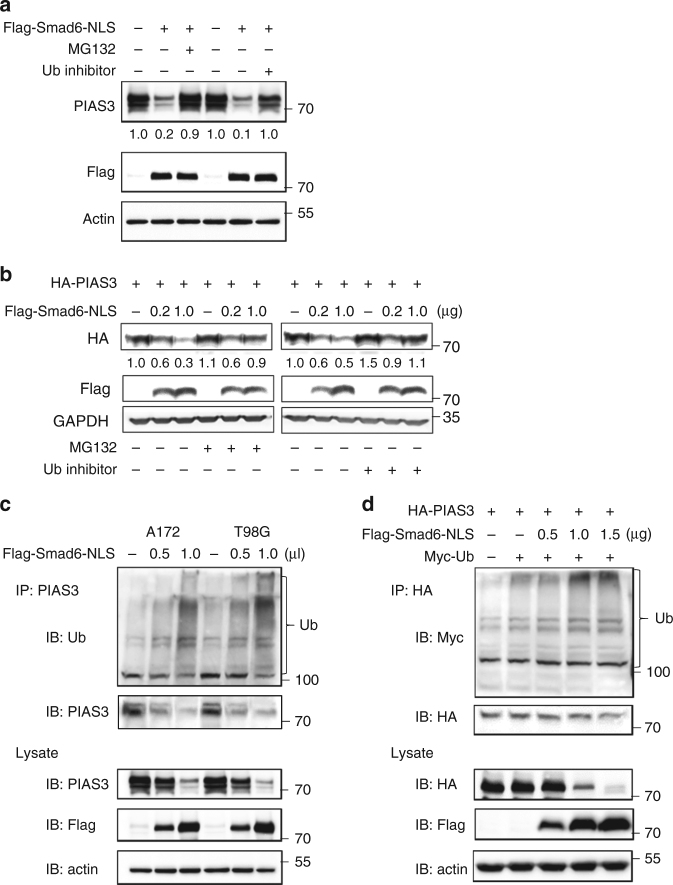
Smad6 negatively regulates PIAS3 via the ubiquitin-proteasome pathway. **a** Nuclear-Smad6 OE decreased endogenous PIAS3 levels in A172 cells through the ubiquitin-proteasome pathway. MG132 (10 μM) or Ub inhibitor (20 ng ml^−1^) was added 12 h before collecting. **b** Nuclear-Smad6 decreased exogenous PIAS3 through the ubiquitin-proteasome pathway. HA-PIAS3 plasmid (600 ng) was co-transfected with increasing amounts of nuclear-Smad6 construct (0, 0.2, 1.0 μg per well in a 6-well plate) into 293T cells. MG132 or Ub inhibitor was added 12 h before harvest. **c**,**d** Nuclear-Smad6-mediated ubiquitination and proteasomal degradation of endogenous and of exogenous PIAS3. After infected (**c**) or transfected (**d**) with indicated constructs for 36 h, cells were treated with 10 μM MG132 for 4 h before collecting. Immunoprecipitation (IP) was performed using the indicated antibodies

Surprisingly, nuclear-Smad6 OE induced an obvious increase of *PIAS3* mRNA in A172 and T98G cells (Supplementary Figure 10a). The positive correlation of these two molecules at mRNA level was further confirmed in patient-derived glioma tissues (Supplementary Figure 10b) and public database analysis (Supplementary Figure 10c, d). These results negated the possibility of Smad6-mediated transcriptional inhibition of *PIAS3*. As previous research showed that PIAS3 is post-transcriptionally repressed in glioma cell lines and regulated by proteasomal degradation^8^, we speculated that the ubiquitin-proteasome pathway is the underlying mechanism of nuclear-Smad6-mediated PIAS3 downregulation. To test this, A172 cells were forced to overexpress Flag-Smad6-NLS mediated by adenovirus. We observed that inhibiting ubiquitin (Ub) or proteasome activity by specific inhibitors partially prevented nuclear-Smad6-induced endogenous PIAS3 downregulation (Fig. 5a), which was further confirmed in 293T cells expressing exogenous PIAS3 (Fig. 5b). Moreover, a ubiquitination assay showed that nuclear-Smad6 induced endogenous (Fig. 5c) and exogenous (Fig. 5d) PIAS3 degradation via the ubiquitin-proteasome pathway in a dose-dependent manner. Collectively, these results indicate that nuclear-Smad6 decreases PIAS3 stability by promoting its ubiquitination.

### MH2 domain is essential for Smad6 interaction with PIAS3

**Fig. 6:**
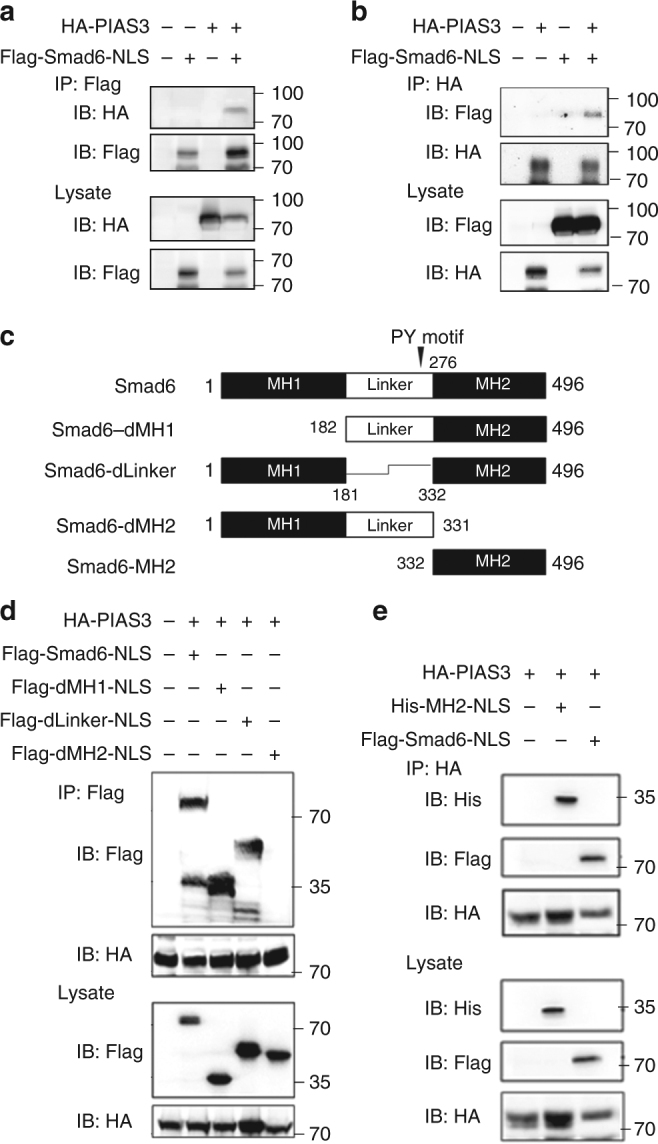
MH2 domain is essential for the interaction of Smad6 with PIAS3. **a** Smad6 bound to PIAS3. HA-PIAS3 was expressed in 293T cells with or without Flag-tagged nuclear-Smad6 construct (Flag-Smad6-NLS), followed by IP with anti-Flag antibody. **b** PIAS3 interacted with Smad6. HA-PIAS3 was expressed in 293T cells with or without Flag-Smad6-NLS, followed by IP with anti-HA antibody. **c** Schematic diagram of Smad6 and its deletion mutants. **d** Mapping of the domain of Smad6 for its interaction with PIAS3. 293T cells were co-transfected with HA-PIAS3 and Flag-tagged nuclear-Smad6 deletion mutants, followed by IP with anti-HA antibody and IB analysis with indicated antibodies. **e** MH2 of Smad6 could bind to PIAS3. 293T cells were co-transfected with HA-PIAS3 and His-MH2-NLS or Flag-Smad6-NLS and followed with an IP analysis using the indicated antibodies

Given that PIAS3 could interact with Smad2, 3 and 4^25^, we speculated that PIAS3 might interact with Smad6 as well. Immunoprecipitation (IP) experiments determined that the endogenous interaction between Smad6 and PIAS3 exists in glioma cells (Supplementary Figure 11). To further address this interaction, we examined it in 293T cells. 293T cells were co-transfected with HA-PIAS3 and Smad6 plasmids. As HA-PIAS3 was predominately expressed in the nuclei of 293T cells (Supplementary Figure 12) and showed less co-localization with Flag-Smad6, the nuclear-expressing Smad6 (Flag-Smad6-NLS) and its nuclear mutants were used for the subsequent co-transfection. An IP assay suggested the interaction between PIAS3 and nuclear-Smad6 (Fig. 6a). An in vitro glutathione *S*-transferase (GST) pull-down assay further revealed that the GST-Smad6 fusion protein was able to pull-down HA-PIAS3 (Supplementary Figure 13, left panel), indicating that Smad6 directly interacts with PIAS3. Reciprocal IP with anti-HA (Fig. 6b) and pull-down assay with GST-PIAS3 (Supplementary Figure 13, right panel) further confirmed the specific PIAS3-Smad6 interaction. To identify the interaction region of Smad6 with PIAS3, we performed IP experiments using truncated nuclear mutants of Smad6 (Fig. 6c). Compared with full-length Smad6, Smad6 with MH1 deletion (dMH1, residues 182–496) or Linker domain deletion (dLinker, residues 1–181 ~ 332–496) exhibited similar binding ability as Smad6 to PIAS3, whereas MH2 deletion (dMH2, residues 1–331) completely abolished the binding ability of Smad6 to PIAS3 (Fig. 6d). In contrast, a MH2 domain construct, His-MH2-NLS, could bind to PIAS3 (Fig. 6e), indicating the MH2 domain is essential for Smad6 interaction with PIAS3. To determine which domain of PIAS3 mediates its interaction with Smad6, we constructed series of truncated nuclear-PIAS3 mutants (Supplementary Figure 14a). IP experiments showed that truncated PIAS3 mutants N, C, and dRing failed to bind Smad6. However, the Ring-expressing construct, HA-Ring-NLS, retained the binding affinity for Smad6 (Supplementary Figure 14b) and Smad6 MH2 domain (Supplementary Figure 14c). These results indicate that the Ring domain (residues 303–380) of PIAS3 is responsible for its specific interaction with Smad6.

### MH2 is essential for Smad6-mediated PIAS3 degradation

**Fig. 7:**
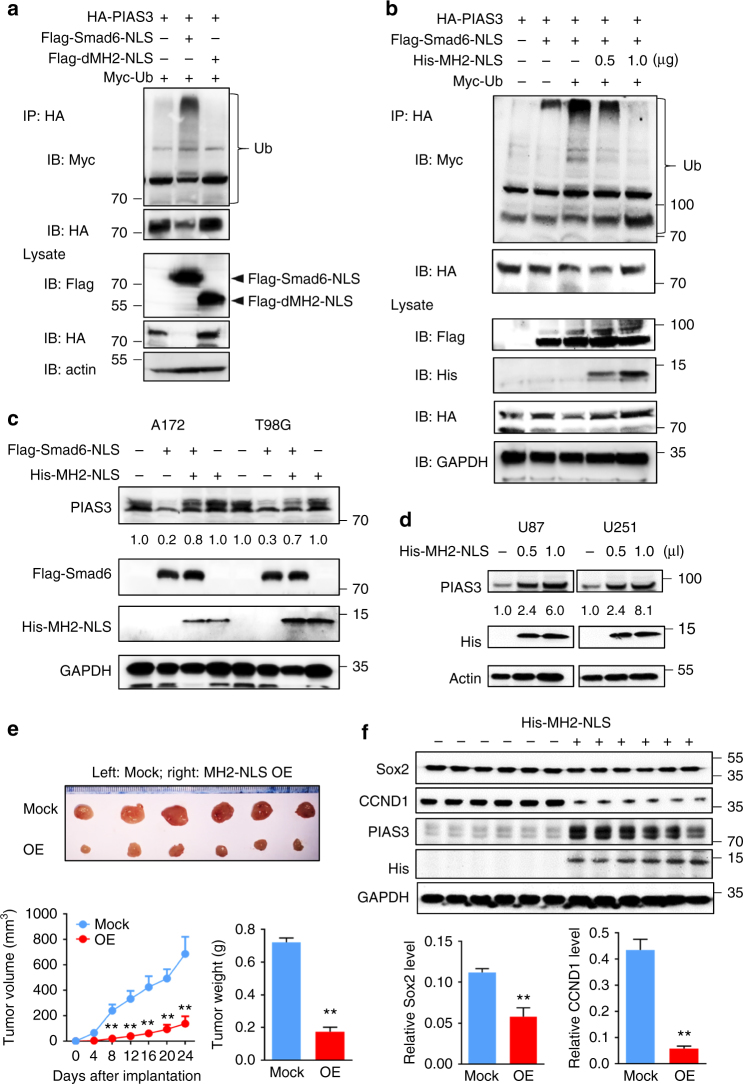
MH2 domain is essential for Smad6-mediated PIAS3 ubiquitination and proteasomal degradation. **a** Smad6-MH2 deletion mutant failed to induce PIAS3 ubiquitination and degradation. 293T cells were transfected with expression constructs in the indicated combinations. **b** Nuclear-MH2 antagonized the ubiquitination and degradation of PIAS3 induced by nuclear-Smad6 in a dose-dependent manner. 293T cells were transfected with expression constructs in the indicated combinations and concentrations (0, 0.5, 1.0 μg per well in a 6-well plate). **c** Nuclear-MH2 rescued nuclear-Smad6 induced PIAS3 downregulation in A172 and T98G cells. **d** Nuclear-MH2 increased endogenous PIAS3 levels in U87 and U251 cells. **e** Nuclear-MH2 inhibited tumor growth in nude mice. U87 cells with nuclear-MH2 OE or Mock were used for tumor formation in a nude mouse xenograft model, and mean tumor volumes and average tumor weight of xenograft tumors were measured (*n* = 6). **f** MH2 introduction increased PIAS3 expression and impaired STAT3 downstream genes (*CCND1* and *SOX2*) expression in xenograft tumors (*n* = 6). Data were represented as means ± SD and analyzed using two-tailed Student’s *t*-test. **P < 0.01

Next, we found that MH2-deleted Smad6 failed to induce PIAS3 ubiquitination and degradation in 293T cells (Fig. 7a). We then analyzed whether the nuclear-MH2 construct, His-MH2-NLS, could act as a dominant-negative mutant to inhibit full-length Smad6 activity using an in vitro ubiquitination experiment. We found that introduction of His-MH2-NLS inhibited nuclear-Smad6-induced PIAS3 ubiquitination and degradation in a dose-dependent manner (Fig. 7b). It rescued endogenous PIAS3 expression inhibited by nuclear-Smad6 in A172 and T98G cells (Fig. 7c). Meanwhile, His-MH2-NLS has no effect on endogenous PIAS3 levels in A172 and T98G cells, suggesting that MH2 alone is insufficient to execute Smad6-induced PIAS3 degradation. Most importantly, His-MH2-NLS increased endogenous PIAS3 expression in U87 and U251 cells (Fig. 7d). As PIAS3 acts as a tumor repressor in GBM, we further determined the effects of MH2-mediated Smad6–PIAS3 interaction on glioma biological phenotypes. We established stable nuclear-MH2-expressing U87 and U251 cell lines and investigated its potential tumor suppressive functions. The tumors stably expressing nuclear-MH2 grew more slowly in vivo (Fig. 7e). Importantly, the expression of PIAS3 was rescued in tumors with MH2 OE. As a result, these tumors expressed lower levels of STAT3 downstream genes (*CCND1* and *SOX2*) than control tumors (Fig. 7f). Thus, these observations suggest that the MH2 domain of Smad6 is essential for Smad6-induced PIAS3 degradation and promotion of STAT3 activity.

In addition, we analyzed whether the Ring domain can also act as a competitive inhibitor to prevent Smad6-induced PIAS3 degradation. As shown in Supplementary Figure 15a, nuclear-expressing Ring dose dependently impaired Smad6-mediated PIAS3 ubiquitination and degradation in 293T cells. In A172 and T98G cells, nuclear-Smad6 introduction resulted in a reduction of PIAS expression, whereas nuclear-Ring impaired this reduction, suggesting that this Ring construct could be a protector for PIAS3 by antagonizing nuclear-Smad6-induced PIAS3 degradation. Surprisingly, we noted that nuclear-Ring triggered a reduction of endogenous PIAS3 in A172 and T98G cells even without Smad6 OE (Supplementary Figure 15b, c). Moreover, nuclear-Ring did not increase endogenous PIAS3 expression as observed with MH2 OE in U87 and U251 cells (Supplementary Figure 15d). These findings suggest that the Ring domain mediated PIAS3 interaction with Smad6 and exhibits inhibitory function only in cells with excessive nuclear-Smad6 expression, whereas Ring itself is inhibitory to endogenous PIAS3 expression and thus promotes tumor development.

### Recombinant MH2 protein is a potential therapy for glioma

**Fig. 8:**
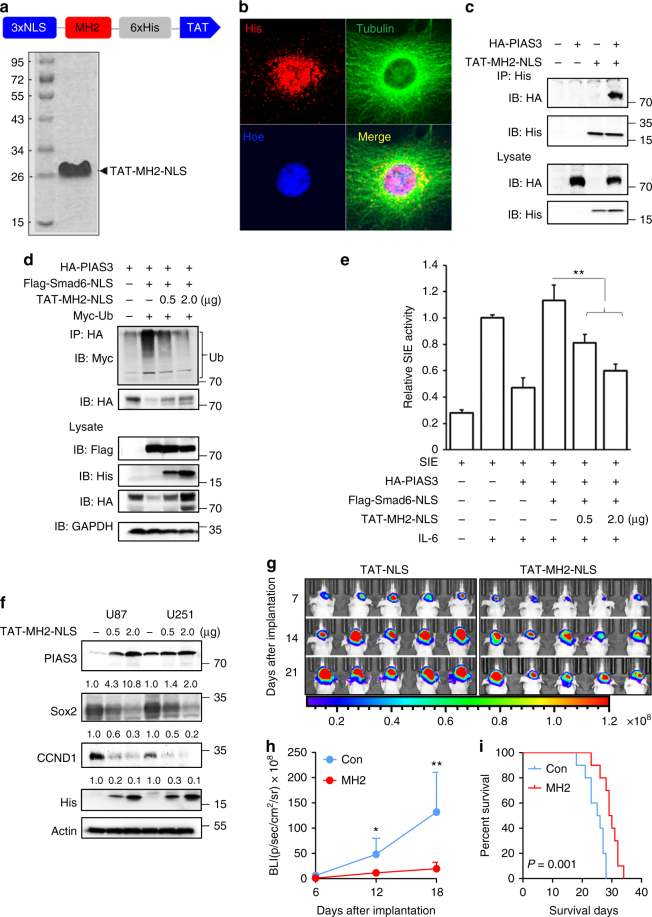
Cell-penetrating MH2 protein could be a potential treatment for glioma. **a** Schematic representation of the TAT-MH2-NLS construct (upper). TAT-MH2-NLS protein was expressed in 293T cells and purified as described in the Methods section (lower). **b** Nuclear accumulation of TAT-MH2-NLS protein. 293T cells were treated with 0.5 μg ml^−1^ TAT-MH2-NLS for 6 h before fixation. Immunofluorescence (IF) staining was performed with anti-His (red) and anti-β-tubulin (Tubulin, green) antibodies and cell nuclei were stained with Hoechst (Hoe, blue). **c** TAT-MH2-NLS protein bound to PIAS3. 293T cells were transfected with HA-PIAS3 and treated with 1.0 μg ml^−1^ TAT-MH2-NLS and followed by IP analysis with anti-His antibody. **d** TAT-MH2-NLS inhibited Smad6-induced PIAS3 ubiquitination and degradation. 293T cells were transfected with indicated constructs and incubated with indicated dose of TAT-MH2-NLS protein. After 24 h treatment, cells were treated with 10 μM MG132 for 4 h before collecting. IP was performed using anti-HA antibody followed by IB with indicated antibodies. **e** TAT-MH2-NLS protein antagonized STAT3-mediated transcriptional activation. Luciferase assay of SIE activity was performed in HEK293 cells transfected with HA-PIAS3 and treated with indicated amounts of TAT-MH2-NLS (*n* = 3). **f** TAT-MH2-NLS increased endogenous PIAS3 expression in U87 and U251 cells. **g**, **h** TAT-MH2-NLS inhibits GBM tumor growth. The tumor-bearing mice were treated with TAT-NLS (Con) or TAT-MH2-NLS (MH2). Bioluminescent images (**g**) and quantification (**h**) of xenografts derived from U87 implantation (*n* = 10). **i** TAT-MH2-NLS treatment prolongs the animal survival. Kaplan–Meier survival curves of mice implanted with U87 cells (Log-rank *χ*^2^ = 10.88, *P* < 0.001. *n* = 10). Data were represented as means ± SD and analyzed using two-tailed Student’s *t*-test in **e**, **h**. *P < 0.05, **P < 0.01

In light of our findings, targeting Smad6 MH2 may present a novel therapeutic approach in glioma. The transactivating transcription factor protein transduction domain (TAT-PTD) peptide derived from the HIV TAT protein can deliver macromolecular cargo into various cell types^26^. To assess the potential application value of MH2, we constructed a cell-penetrating MH2-NLS protein fused to TAT-PTD (Fig. 8a). Immunostaining showed that TAT-MH2-NLS fusion protein efficiently accumulated within cells, with most reaching nuclear compartments (Fig. 8b). IP analysis showed that TAT-MH2-NLS could bind to PIAS3 (Fig. 8c) and antagonized Smad6-induced PIAS3 ubiquitination and degradation in a dose-dependent manner (Fig. 8d). As a result, TAT-MH2-NLS retained the effect of PIAS3 in the SIE reporter assay through counteraction with nuclear-Smad6 (Fig. 8e). Moreover, TAT-MH2-NLS treatment increased endogenous PIAS3 expression and reduced expression of STAT3 downstream genes (*CCND1* and *SOX2*) in U87 and U251 cells (Fig. 8f). These data suggested that this recombinant protein exhibits bio-active tumor-inhibitory properties. To address the impact of TAT-MH2-NLS on tumor propagation, we examined its effects on glioma tumorigenesis. U87 cells expressing luciferase were implanted into mouse brains. The mice were injected intraperitoneally (i.p.) with TAT-MH2-NLS (MH2) or TAT-NLS (Con) once every 2 days from the second day after implantation. Bioluminescent analyses showed that TAT-MH2-NLS potently inhibited tumor growth in situ (Fig. 8g, h). Mice treated with TAT-MH2-NLS exhibited extended survival relative to those treated with control protein (Fig. 8i). To confirm the findings in established cell lines, we investigated the effect of TAT-MH2-NLS protein on tumor growth using patient-derived glioma cells. The patient-derived glioma cells (T06) expressing high Smad6 and low PIAS3 were implanted into mouse brains. As shown in Supplementary Figure 16a and b, TAT-MH2-NLS treatment potently inhibited tumor growth and extended mice survival. To investigate whether TAT-MH2-NLS affected the expression of PIAS3 and STAT3 target genes, we performed immunofluorescent analyses in T06-derived xenografts and revealed that PIAS3 was significantly increased in the xenografts treated with MH2 protein (Supplementary Figure 16c). In contrast, the expression of Ki67 and STAT3 target genes (*CCND1* and *SOX2*) were significantly reduced in the MH2-treated xenografts (Supplementary Figure 16d–f), indicating a therapeutic effect of TAT-MH2-NLS protein for glioma. Collectively, these data demonstrate that TAT-MH2-NLS administration appears to be a promising therapy strategy for glioma.

### Smurf1 is involved in Smad6-mediated PIAS3 degradation

**Fig. 9:**
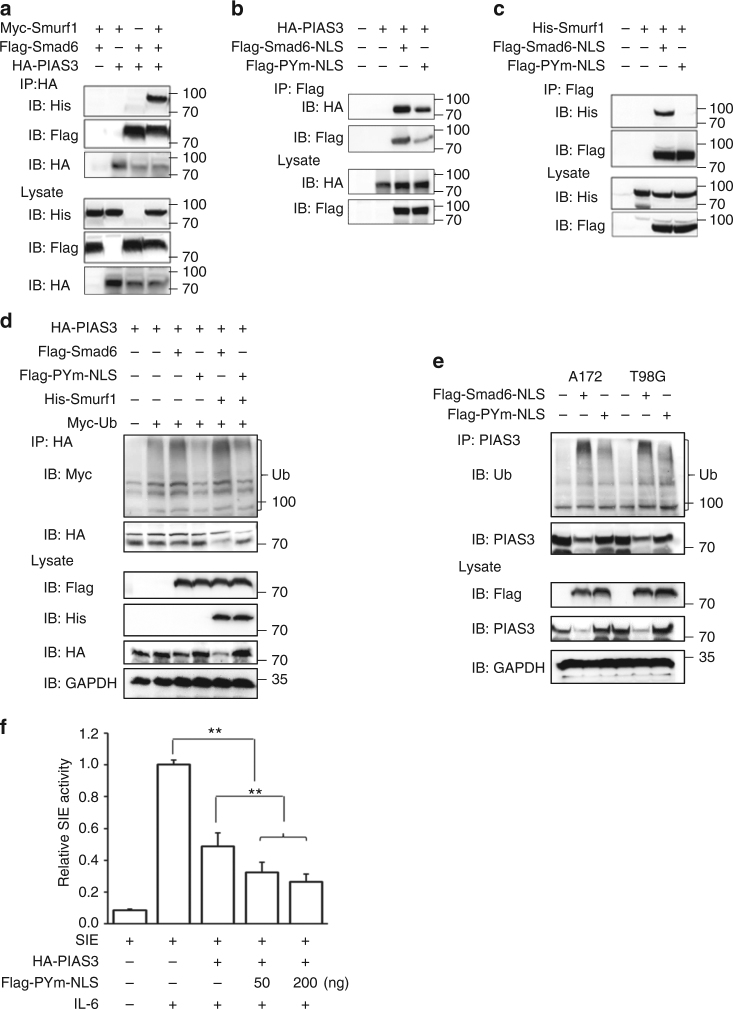
Smurf1 is required for Smad6-induced PIAS3 ubiquitination and degradation. **a** Smurf1 bound to Smad6 and was present in the Smad6–PIAS3 complex. 293T cells were transfected with expression constructs in the indicated combinations followed by IP analysis. **b** Smad6 with PY mutation (PYm) bound to PIAS3. **c** Smad6 with PY mutation failed to bind to Smurf1. **d** PYm abolished the effect of Smad6 on the ubiquitination and degradation of exogenous PIAS3 in 293T cells. **e** PYm abolished the effect of Smad6 on the ubiquitination and degradation of endogenous PIAS3 in A172 and T98G cells. **f** PYm failed to rescue IL-6-dependent SIE activity that was inhibited by PIAS3. Luciferase assay of SIE activity was performed in HEK293 cells transfected with HA-PIAS3 and indicated amounts of nuclear-Smad6 PYm (0, 50, and 200 ng) (*n* = 3). IL-6 (25 ng ml^−1^) was added 6 h before assay. Data were represented as means ± SD and analyzed using two-tailed Student’s *t*-test in **f**. **P < 0.01

Smurf1, an E3 ubiquitin-protein ligase, has been reported as crucial for the inhibitory activity of Smad6 and Smad7^27,28^. Smad6 could recruit Smurf1 to trigger the degradation of associated proteins^27,29^. Therefore, we speculated that Smad6 also recruits Smurf1 to facilitate PIAS3 ubiquitination and degradation. First, we determined whether Smurf1 could be involved in the Smad6–PIAS3 interaction complex. As shown in Fig. 9a, Smurf1 was detected in the PIAS3 immunoprecipitated complex only in the presence of Smad6. Smurf1 has been shown to interact with the PY motif (PPxY) of Smad6^29^. We constructed a Smad6 PPXY mutant (PPxA, PYm) and demonstrated that it did not affect the Smad6–PIAS3 interaction (Fig. 9b). However, this mutant failed to bind Smurf1 (Fig. 9c), suggesting that Smurf1 is recruited to PIAS3 through interaction with Smad6 via the PY motif. Further IP experiments showed that the ubiquitination and degradation of PIAS3 induced by nuclear-Smad6 were enhanced by Smurf1, whereas PYm abolished Smad6 influence on the ubiquitination and degradation of exogenous and endogenous PIAS3 even with the involvement of Smurf1 (Fig. 9d, e). Interestingly, PYm not only failed to rescue IL-6-dependent SIE activity that was inhibited by PIAS3, but also enhanced the inhibitory effect of PIAS3 on SIE activity (Fig. 9f). Taken together, Smurf1 appears to be recruited to PIAS3 and facilitates its degradation through interaction with the PY motif of Smad6.

## Discussion

**Fig. 10:**
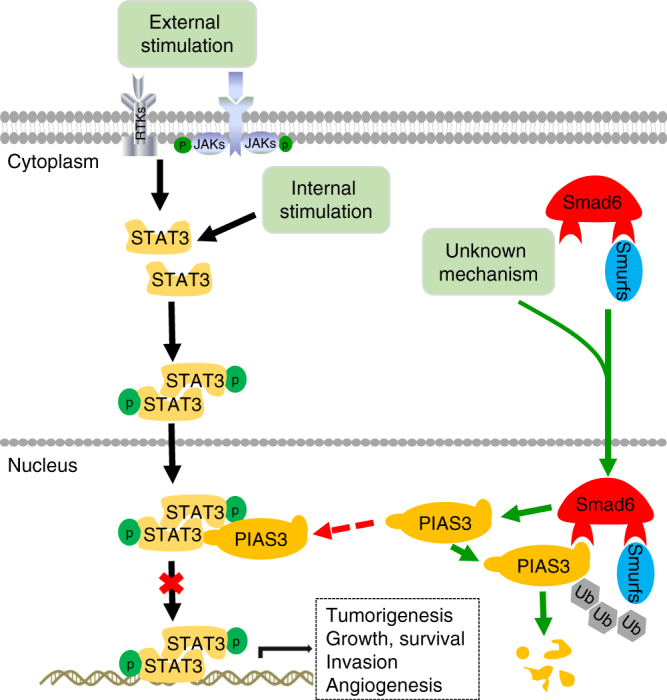
A putative working model of Smad6–PIAS3–STAT3 regulation axis. STAT3 is activated in an external stimulation-dependent manner (IL-6, EGF, or other cytokines) or external stimulation-independent manner through phosphorylation of the STAT3 tyrosine residue. Activated STAT3 forms homodimers or STAT3/STAT1 heterodimers and translocates to the nucleus to function as a transcription factor. PIAS3 binds activated STAT3 and prevents its binding to DNA, resulting in the downregulation of STAT3-dependent transcription. Undetermined signals induce Smad6 nuclear translocation. Nuclear-Smad6 indirectly activates STAT3-dependent transcription through promoting the ubiquitination

In contrast to the transient STAT3 activation in normal cells, constitutive or excessive active STAT3 is frequently in many types of tumors and promotes tumorigenesis^30^. Abolishing STAT3 activity may be an attractive cancer therapeutic strategy, while the underlying mechanisms of STAT3 activation deregulation in cancers is largely unknown. PIAS3 is an endogenous inhibitor of STAT3 through prohibiting its DNA binding, and the loss of PIAS3 in cancers contributes to enhanced STAT3 activity and subsequent tumor growth, as well as impacts patient survival, identifying PIAS3 a promising therapeutic target^12,31–33^. However, mechanisms regulating PIAS3 downregulation in human cancers have not been comprehensively studied^34,35^. Here we provide evidence to connect elevated Smad6 and low PIAS3 to constitutive STAT3 activity in gliomas. A novel mechanism is revealed showing that nuclear-Smad6 acts as a novel PIAS3-interacting protein and antagonizes PIAS3-mediated STAT3 transcriptional activity inhibition by accelerating PIAS3 ubiquitination and degradation (Fig. 10).

PIAS3 acts as a “buffer” to control STAT3 activity at an appropriate level and loss of PIAS3 allows STAT3 activation to sustain transcription of genes that control tumorigenesis^12^. PIAS3 does not modulate the phosphorylation of STAT3, but regulates its ability to bind target DNA^12,36^. The present study reveals a mechanism underlying PIAS3 loss in gliomas and explains the previous finding that PIAS3 loss in gliomas at protein level, and not at mRNA level^12^. Our data reveal a novel Smad6–PIAS3–STAT3 axis in gliomas, contributing to our detailed understanding the importance of the Smad6–PIAS3 signaling in regulating gliomagenesis. In addition to its regulation on STAT3, PIAS3 exhibits anti-tumorigenic roles via other mechanisms, including regulating nuclear factor-κB, PI3K/AKT, and TGFβ pathways^11,14,25,37^, reducing the activity of transcription factors such as MITF, Oct4, and others^38,39^. As a SUMO (small ubiquitin-like modifier)-E3 ligase, the anti-tumor functions of PIAS3 are also associated with sumoylation enhancement and degradation of oncoproteins^40–42^, as well as a promoting regulation of p53 and ATR by inducing p53 sumoylation^43–45^. The complicated mechanisms of PIAS3 in tumorigenesis indicate that besides STAT3, other potential downstream pathways mediating the cancer-promotion of Smad6–PIAS3 axis in gliomas still need to be studied. Nevertheless, our data are an important step for understanding PIAS3 loss in gliomas and identifying new targeted therapeutic strategies. Surprisingly, the present study also showed a positive correlation between SMAD6 and PIAS3 at mRNA level in gliomas. We speculate that Smad6 may act as a promoting factor, which directly or indirectly regulates PIAS3 transcription, although whether these are parallel events cannot be ruled out. In addition, the contradictory expression patterns of PIAS3 at mRNA and protein levels may be explained by a feedback mechanism that PIAS3 protein degradation induces a responsive PIAS3 transcriptional activation.

Although prior studies suggest that Smad6 is associated with tumorigenesis^18,21,46^ and metastasis^34^, little is concerning the expression, function, and mechanism of Smad6 in the glioma. The present study found that Smad6, especially nuclear-Smad6, was highly expressed in gliomas, and elevated expression of Smad6 correlated with poor survival. Functional experiments identified a key role for nuclear-Smad6 in promoting tumor sphere formation, proliferation and tumorigenesis of GBM cells. These findings, combined with those obtained from clinical tissue analysis represent strong evidence that nuclear-Smad6 is a key factor in glioma pathogenesis. As a negative regulation factor of BMP and TGFβ signaling, Smad6 mediates multiple signaling pathways and interacts with multiple proteins, such as GR, Runx2, and Tbx6^27,47,48^. These data indicate that the tumor promotion of Smad6 in gliomas is not limited to the PIAS3/STAT3 mechanism. It is therefore necessary to further investigate and uncover its functions and underlying mechanisms of action. Although determined to be a nuclear protein, Smad6 was also found to be expressed in cytoplasm in established GBM cell lines. Interestingly, our ongoing study revealed that ectopic Smad6 is predominately expressed in cytoplasm and can act as a tumor suppressor in inhibiting migration, invasion, and metastasis. Moreover, Smad6 has also been linked to reduced cell growth and overall survival in breast and oral cancers^34,35^. These results further reflect its complicated functions in human cancers, depending on various conditions, such as different cell types and subcellular distribution. It also raises new questions into the causes of different subcellular distributions of Smad6.

Although PIAS3 is closely associated with STAT3 and Smad6 interacts to PIAS3, we did not observe direct interaction between Smad6 and STAT3 in glioma cells. It suggests that Smad6–PIAS3 interaction occurs separately from the STAT3–PIAS3 complex. We speculate that in normal conditions, Smad6 transiently binds PIAS3 in nuclei and regulates the PIAS3–STAT3 complex at an appropriate level and time, thus precisely maintaining STAT3 at a reasonable level; however, in cancer-inducing conditions, increased Smad6–PIAS3 interaction destroys the regulation balance and results in aberrant STAT3 transcriptional activation, as well as other oncoproteins deregulation.

An important discovery of the present study is that Smad6 MH2 domain is required for the Smad6–PIAS3 interaction. It sheds light on targeting MH2 as a novel therapeutic approach for glioma. TAT-MH2-NLS protein could function as an antagonist to inhibit Smad6-induced PIAS3 ubiquitination and degradation. Moreover, this protein exhibits significant anti-tumor effects in tumor-bearing animal models. Considering the importance of MH2 in mediating the interaction of Smad6 with other factors^16,27,49^, further research is necessary to fully evaluate and optimize TAT-mediated targeting of the Smad6 MH2 domain for cancer therapy. As Smad6 is a key protein in regulating glioma stem-like cell initiation and self-renewal, an additional concern is that MH2 protein might have inhibitory effects on cancer stem cells and reverse chemoradiotherapy resistance.

The present study also reveals that Ring domain is essential for the interaction of PIAS3 with Smad6. Although the Ring construct exhibits its protection on PIAS3 only with excessive nuclear-Smad6 expression, it triggers a reduction of endogenous PIAS3 in native GBM cells and thus demonstrates a cancer-promoting function. As a SUMO-E3 ligase, PIAS3 could autosumoylate and modulate stability of itself^50^. Ring domain is essential for the SUMO-E3 ligase activity of PIAS3^25,51,52^ and its regulation on target proteins^16,53^. It suggests that Ring is the vital domain for PIAS3 autosumoylation and the introduction of Ring destabilizes PIAS3 via impairing its autosumoylation and enhancing ubiquitination^54^. Moreover, the Ring-induced reduction of PIAS3 suggests an additional underlying mechanism mediating PIAS3 loss in cancers. It also supposes that a self-regulatory balance of PIAS3 exists under normal physiologic conditions, and once the balance is disrupted, aberrant cell proliferation and tumorigenesis can occur. Consequently, the Ring construct might be an efficient tool to investigate underlying regulation mechanisms.

In addition to general alterations in Smad posttranslational modifications and functions, reduced Smurf1 expression and prevention from interacting with other Smads are known outcomes of Smad6-mediated inhibition of cellular TGF/BMP signaling^15,55,56^. In light of this evidence, and the fact that Smad6 has been reported to recruit Smurf1 to promote degradation of associated proteins^27,29^, we examined whether Smurf1 is recruited by Smad6 to mediate PIAS3 ubiquitination and breakdown. Our results showed that ectopic Pym expression failed to rescue IL-6-dependent SIE activity that was inhibited by PIAS3 and instead enhanced overall PIAS3 inhibition of SIE activity. Although Smurf1 was unable to bind directly to PIAS3, we determined that the Smad6 PY motif was critical for the interaction of Smurf1 to induce PIAS3 degradation.

In conclusion, our results highlight key roles of nuclear-Smad6 as a new regulator of STAT3 through PIAS3 interaction in gliomas. This study also uncovers new questions and possibilities concerning the role and mechanistic activity of inhibitory SMADs in various cancers. Given that Smad6 interacts and regulates many other proteins, future studies will aim to investigate additional mechanisms involved in tumorigenesis.

## Methods

### Clinical samples and IHC

Tissue arrays and IHC staining were conducted as described previously^21^. Tissue microarray chips containing a total of 149 case samples (142 gliomas and 7 normal brain tissues) with follow-up data were from affiliated hospital. All patient information was obtained and used in accordance with approved protocols from the institutional review boards of the participating institutions. The clinical characteristics of the cohort were described in Supplementary Data 1. Briefly, tissue slides were incubated with rabbit anti-Smad6 antibody (1:4000, Sigma-Aldrich, St. Louis, MO) or rabbit anti-PIAS3 antibody (1:500, Cell Signaling, Inc., Danvers MA), anti-rabbit secondary antibody from Zymed Systems (Invitrogen, Carlsbad, CA) and 3,3’-diaminobenzidine to visualize IHC labeling. Slides were counterstained lightly with crystal violet. Normal rabbit IgG was used to confirm the specificity of the IHC labeling. Smad6 and PIAS3 expression were scored semi-quantitatively on the basis of an established immunoreactivity scoring (IRS) system^57,58^. Briefly, IRS covers a range of 0–12 as a product of multiplication of proportion score (0–4) and staining intensity score (0–3). The proportion score represents the percentage of positive cells (0: no positive cells; 1: < 10% of positive cells; 2: 10–50% positive cells; 3: 51–80% positive cells; 4: > 80% positive cells). The intensity score represents the average intensity of staining (0: no staining; 1: yellow, 2: claybank; and 3: tawny). The histologic slides were concurrently checked and scored by two blinded pathologists. The mean IRS core was considered as the final IRS (Supplementary Data 1).

### Online cancer database analysis

An online glioma database (GSE4290) containing 157 patients and 23 non-tumor controls with emphasis on Smad6 gene expression was accessed through NCBI (http://www.ncbi.nlm.nih.gov/geo/tools/profileGraph.cgi?ID = GDS1962:209886_s_at). A large cohort survival analysis was performed using TCGA GBM database from Project Betastasis (http://www.betastasis.com). The clinical characteristics of the cohort and *SMAD6*/*PIAS3* expression data are described in Supplementary Table 3 and Supplementary Data 2.

### Cell culture

Human glioma cell lines, U87, U251, T98G and A172, and human embryonic kidney cell line, 293T, were obtained from the Cell Bank of Type Culture Collection of the Chinese Academy of Sciences (Shanghai, China) and cultured in Dulbecco’s modified Eagle’s medium (DMEM) with 10% fetal bovine serum (FBS; Gibco, Carlsbad, CA). These cells were characterized by Genewiz, Inc. (China) using short tandem repeat markers and were confirmed to be mycoplasma-free (latest tested in 2016). For patient-derived tumor cell culture, fresh brain glioma tissues were collected within 30 min after tumor resection, washed, minced, and enzymatically dissociated. Tumor cells were resuspended and cultured in DMEM/F12 medium containing 10% FBS. After identification by immunostaining using Nestin and GFAP antibodies, the second or third passage of cells was used for tumor xenografts and western blot assay.

### Vector construction and transduction

A lentiviral *SMAD6* short hairpin RNA (shRNA) was purchased from Genechem (Shanghai, China). The shRNA sequence targeting human *SMAD6* complementary DNA was: 5′-CCGAUUCCACAUUGUCUUA-3′. A scrambled shRNA was included as a negative control (NC). The target sequence was inserted into GV248 lentiviral vector (Genechem). The small interfering RNA (siRNA) sequence targeting human *SMAD6* and *STAT3* were purchased from Dharmacon and Cell Signaling, Inc., respectively. The siRNA sequences targeting human *PIAS3* were: 5′-GGAGCCAAAU GUGAUUAUAUU-3′; 5′-GACA GAGAGUCAGCACUAUUU-3′, and synthesized by Ribobio (Guangzhou, China). Their scrambled sequences served as NCs. Transfection was performed using X-tremeGENE siRNA transfection reagent (Roche, Mannheim, Germany). For western blot analysis, cell proteins were prepared 72 h post transfection.

Full-length cDNA encoding human *SMAD6* and its serial deletion mutants were amplified by PCR and verified by DNA sequencing. The sequences were cloned into the adenoviral vector GV138 (Genechem) with a Flag-tag. To obtain a nuclear-forced Smad6 construct (Smad6-NLS) or its mutant constructs, 3 × NLS sequence was fused at its N terminus. A DNA fragment encoding Smad6 MH2 was cloned into the pcDNA3.1 plasmid (Invitrogen) with a His-tag to construct a Smad6 MH2-expressing plasmid with 3 × NLS at N terminus. A Flag-tagged PY motif mutation (PPXA, PYm) of the Smad6 plasmid was constructed based on the wild-type Smad6 plasmid. Full-length cDNA encoding human *PIAS3* and its serial deletion mutants were cloned into the adenoviral vector GV366 plasmid (Genechem) with a HA-tag. Tandem repeat of the NLS were fused at N terminus to obtain nuclear-forced PIAS3 deletion mutants. His-tagged Smurf1 and Myc-tagged Ubiquitin (Ub) plasmids were derived from human full length of Smurf1 and Ubiquitin, respectively.

### Recombinant protein purification

The 11 amino acids of TAT-PTD (YGRKKRRQRRR) derived from the HIV TAT protein can deliver macromolecular cargo into various cell types. A cell permeant form of Smad6-MH2 (TAT-MH2-NLS) was generated by fusing the TAT-PTD to its C-terminal and 3 × NLS to its N-terminal using standard molecular cloning techniques and a pcDNA3.1 vector. The TAT-MH2-NLS plasmid was transfected into 293T cells and a stable cell line was established under treatment with G418 (Sigma, St. Louis, MO). Cellular proteins were collected and recombinant proteins were affinity purified according the High Affinity Ni-NTA Resin Kit protocol (Genscript, China). Eluted proteins were dialyzed against 20 mM of HEPES (pH 8.0) plus 150 mmol L^−1^ of NaCl at 4°C and frozen in 10% glycerol at 80 °C. TAT-NLS protein was synthesized directly (Genscript) and used as a control.

### IF labeling

IF staining was performed followed our established protocol. Briefly, the fixed cells or tissue sections were premobilized and blocked with 0.3% Triton X‑100 or 3% normal goat serum in 0.01 M phosphate-buffered saline (PBS) for 30 min at room temperature (RT), followed with an overnight incubation of indicated antibodies at 4 °C. On the following day, the cells or sections were incubated with fluorescein isothiocyanate‑conjugated goat anti‑rabbit and TRITC‑conjugated goat anti‑mouse (1:100; Jackson ImmunoResearch Laboratories, Inc., West Grove, PA, USA) antibodies. Cell nuclei were counterstained with Hoechst 33342 (Invitrogen). The sections were washed, mounted, and examined using the Olympus BX60 light (Olympus, Center Valley, PA, USA) or a laser scanning confocal microscope (Leica Microsystems GmbH, Mannheim, Germany). Primary antiserum omission and normal mouse and goat serum controls were used to confirm the specificity of the immunofluorescent labeling.

### Western blot assays

Standard western blot assays were used to measure protein expression. Antibodies used to determine the indicated protein are shown in Supplementary Table 4. The uncropped western blot images were shown in Supplementary Figure 17.

### Reverse-transcription quantitative PCR

Total RNA from primary glioma tissues and cells was extracted using TRIzol reagent (Invitrogen) according to the manufacturer’s instructions. cDNA was synthesized with the Prime Script RT Reagent Kit (TaKaRa). Quantitative PCR (qPCR) analyses were conducted to quantitate mRNA relative expression using Real SYBR Mixture (CoWin Bioscience, China) on a Lightcycler 480 II instrument (Roche Applied Science), with *GAPDH* as an internal control. Primers used for qPCR are shown in Supplementary Table 5.

### Immunoprecipitation and ubiquitination assays

Before analysis, 293T cells were transiently transfected with various expression plasmids as indicated for 48 h. Cell lysates were prepared in RIPA buffer and IP was performed using indicated antibodies. For the ubiquitination assay, cells were treated with MG132 (Abcam, Cambridge, UK) at a final concentration of 20 μM for 4 h before collecting. PIAS3 ubiquitination and degradation were determined by IP using PIAS3 or HA antibody (targeting HA-PIAS3) followed by western blot analysis with anti-Ub or anti-Myc (targeting Myc-Ub).

### GST pull-down assay

GST or GST-Smad6 protein was bound to glutathione-agarose beads (Pierce, Roclford, IL) and incubated for 2 h with total lysates from HA-PIAS3-expressing 293T cells. The beads were washed three times with GST lysis buffer. Proteins were eluted with 2 × SDS loading buffer and analyzed by western blotting using anti-GST and anti-HA antibodies. Reciprocal GST pull-down with GST-PIAS3 was bound to glutathione-agarose beads and incubated for 2 h with total lysates from Flag-Smad6-NLS-expressing 293T cells and analyzed by western blotting using anti-GST and anti-Flag antibodies.

### ChIP assay

ChIP assays were conducted using a SimpleChIP® Enzymatic Chromatin IP Kit (Cell Signaling) according to the manufacturer’s instructions. In brief, cells were crosslinked with 1% formaldehyde for 10 min at RT. The crosslinking was quenched with 0.1 M glycine and washed three times with ice-cold PBS. Cell pellets were resuspended in 1 ml of lysis buffer containing protease inhibitors on ice for 10 min. Subsequently, the chromatin was digested by Micrococcal Nuclease for 20 min at 37 °C. Nuclear pellets were resuspended in lysis buffer and pulse sonicated. The supernatant samples were collected, diluted in 1 × ChIP buffer and incubated with STAT3 antibody overnight at 4 °C with rotation. Ten microliters diluted sample was used for input. Histone H3 Rabbit antibody and normal Rabbit IgG were used for positive control and NC, respectively. The samples were incubated with Protein G magnetic beads for 2 h at 4 °C with rotation. The antibody/protein G magnetic beads were washed and chromatin supernatants were carefully collected. After reversal of cross-links and DNA purification, the samples were used for quantitative real-time PCR with specific primers. Primers used for the *SOX2* promoter were: 5′-CGTCACATGGATGGTTGTCTATTAACTTGTTCA-3′ and 5′- CTCTCAGTCCTAGTCTTAAAGAGGCAGC-3′; The *CCND1* promoter primers were purchased (Cell Signaling, #12531). ChIP efficiency expressed as a percent of the total input chromatin shown in below: %(ChIP/Total input) = 2^[Ct(Input)−Ct(ChIP)]^ × 100.

### Luciferase assay

To evaluate STAT3 transcriptional activity, we used the reporter construct pGL4.47[Luc2P/SIE/Hygro] (SIE-pGL4.47, Promega, Madison, WI), which contains five copies of SIE that drives luciferase reporter gene luc2P expression. 293T cells were transiently transfected with the plasmids, as indicated. pRL-TKRenilla luciferase plasmid (Promega) was used to maintain a constant total amount of transfected DNA in each well through all experiments. Thirty-six hours after transfection, cells were stimulated with IL-6 (20 ng ml^−1^; Peprotech, Inc., Rocky Hill, NJ) for 6 h. The cells were then collected and assayed for luciferase activity by the Dual Luciferase Reporter Assay System (Promega).

### Cell growth assay and colony formation assay

Cell growth was assessed using the Cell Counting Kit-8 Assay Kit (Biotool, Houston, TX) according to the accompanying protocol. Each experiment was repeated six times. For the colony formation assay, 500–1000 indicated cells were seeded into each well of a six-well plate with soft agar (Agarose; Sigma) and maintained in a medium containing 10% FBS for 14 days. The colonies were fixed with methanol and stained with 0.1% crystal violet; the number of clones was counted using an inverted microscope.

### Tumor sphere formation assay

Tumor sphere formation was analyzed as described previously^59–61^. Briefly, photographs of tumor spheres were taken at indicated time points and sphere diameter was measured. For the secondary sphere formation assays in A172 and T98G, the first spheres derived from normal condition were dissociated, infected with Smad6-NLS adenovirus, and allowed to sphere re-formation. Cells for first and secondary sphere assay from U87 and U251 cells were derived from stable cells with lentivirus-mediated Smad6 KD. At least 10 random fields were analyzed per experiment.

### Tumor xenografts in nude mice

Four weeks old female BALB/c nude mice were purchased from the Shanghai Animal Center, Chinese Academy of Sciences. All mice were assigned randomly. To initiate tumors, 5 × 10^6^ cells in 100 μl of DMEM: Matrigel (8:1, v/v; BD Biosciences, Franklin Lakes, NJ) were injected subcutaneously into the flank of each nude mouse by two blinded technicians. Tumor growth was monitored by measuring tumor diameter every 4 days. To evaluate the tumorigenesis of patient-derived glioma cells, the second passage of cells were used. The glioma-bearing mice were euthanized 3 weeks after implantation. The flank xenografted tumors were then removed and measured weight. The removed tumors were embedded in optimal cutting temperature compound (OCT, Sakura Finetek, Torrance, CA) or processed for western blot analyses. For measuring the tumor inhibition of TAT-MH2-NLS, an intracranial xenograft model was used. Briefly, U87 cells stably expressing the firefly luciferase gene (5 × 10^5^ cells in 5 μl PBS) were stereotactically implanted into the brains of individual mice. Tumor growth was monitored via bioluminescent imaging using IVIS Spectrum system and quantified by Living Image Software. To determine the effect of TAT-MH2-NLS on tumorigenicity, mice were injected i.p. once every 2 days with 10 μg of TAT-MH2-NLS or TAT-NLS from second day after implantation. In additional experiments, mice were stereotactically implanted with patient-derived glioma cells (T06) and injected with 10 μg of TAT-MH2-NLS or TAT-NLS once every 2 days. Twenty days after implantation, some of mice were killed and the brains were removed and embedded in OCT compound. The embedded tissues of xenografted tumors were sectioned and subjected to hematoxylin and eosin or IF staining. All animal care and handling procedures were performed in accordance with the National Institutes of Health’s Guide for the Care and Use of Laboratory Animals and were approved by the Institutional Review Board of Nanjing Medical University. No mice were excluded from scoring. All animal experiments were conducted in a two-blinded manner.

### Statistical analysis

Comparisons were performed using the Kaplan–Meier method with log-rank test, *χ*^2^-test, Spearman’s rank correlation analysis, two-tailed Student’s *t*-test. An *F* test was used to test variance equality. Differences were considered significant when *P* *<* 0.05. SPSS 16.0 package (IBM) and Graphpad prism 5.0 software (GraphPad Software) were used for all statistical analyses and data graphing, respectively.

### Data availability

The authors declare that all data supporting the findings of this study are available within the article and its Supplementary Information files or from the corresponding authors on reasonable request.

## Electronic supplementary material

Supplemtary Information

Description of Additional Supplementary Files

Supplementary Data 1

Supplementary Data 2
