# Supplementary material for: Nuclear Smad6 promotes gliomagenesis by negatively regulating PIAS3-mediated STAT3 inhibition
Source: Nat Commun. 2018 Jun 27;9:2504. doi: 10.1038/s41467-018-04936-9 (PMC6021382; doi:10.1038/s41467-018-04936-9)
Supplement: Supplementary file 1 — Supplemtary Information [file 41467_2018_4936_MOESM1_ESM.pdf]

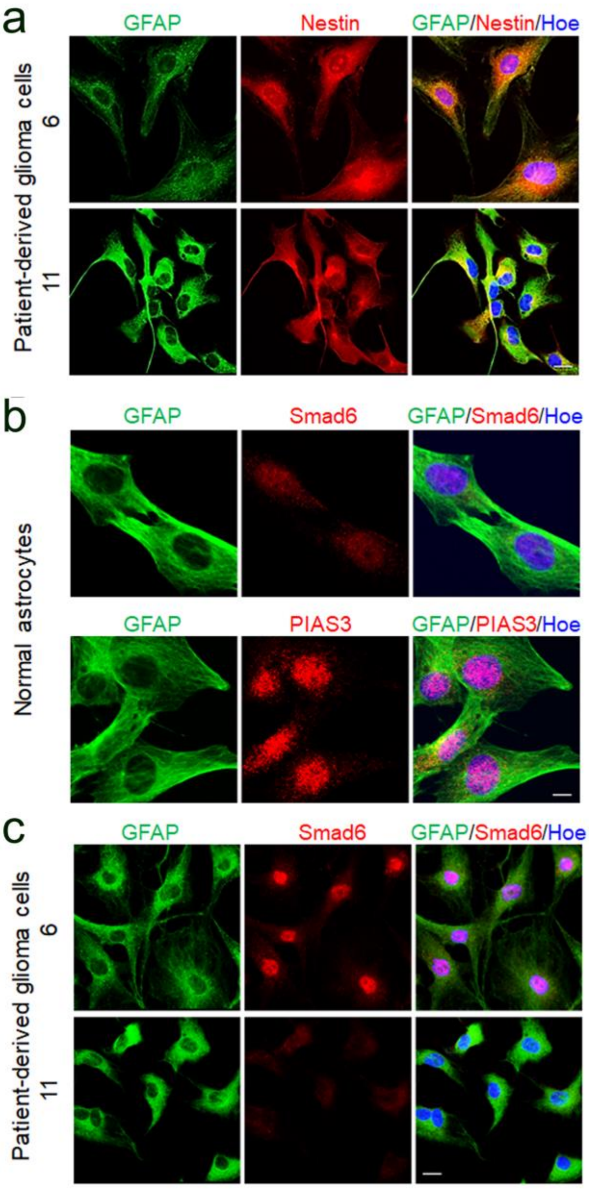

Supplementary Figure 1. Double immunofluorescence (IF) staining of patient-derived glioma cells and normal astrocytes. **(a)** Double IF staining for GFAP and Nestin of cultured patient-derived glioma cells. Hoechst (Hoe) labeled the nuclei. **(b)** Double IF staining for GFAP and Smad6 or PIAS3 of human astrocytes cells. **(c)** Double IF staining for GFAP and Smad6 of patient-derived glioma cells. Scale bars, 20 μm.

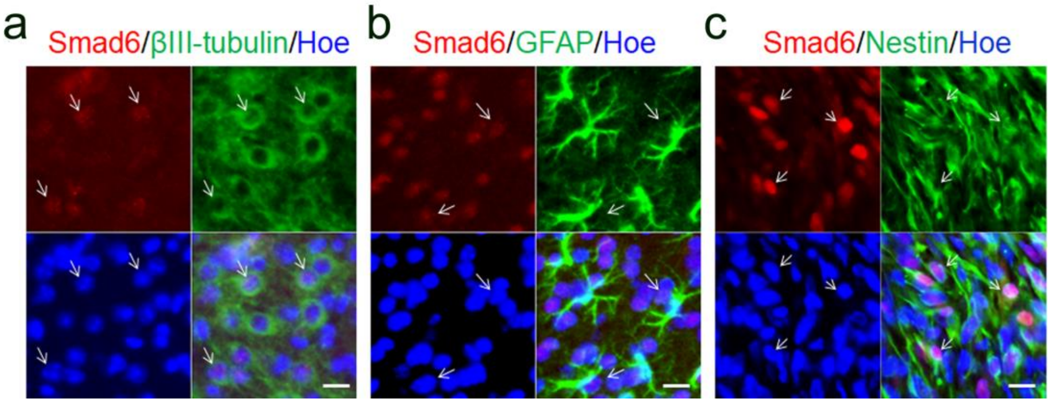

Supplementary Figure 2. Sub-cellular localization of Smad6 in normal human brain and GBM tissue. **(a)** Double IF staining for βIII-tubulin and Smad6 of normal human brain section. **(b)** Double IF staining for GFAP and Smad6 of normal human brain section. **(c)** Double IF staining for Nestin and Smad6 of GBM tissue section. Arrows indicated the representative Smad6 positive cells. Scale bars, 20 μm.

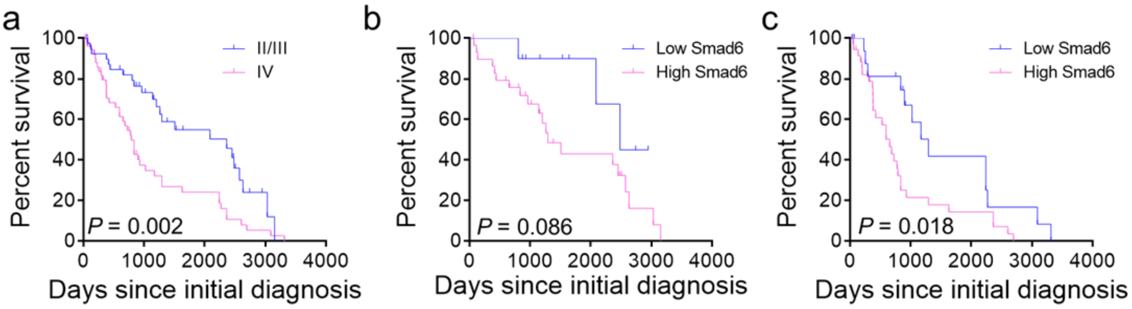

Supplementary Figure 3. Smad6 overexpression in gliomas predicts poor survival. **(a)** Survival analysis of patients from IHC array based on different grades of gliomas. The significance was listed in right panel. **(b,c)** Smad6 overexpression predicts poor survival in different grades of gliomas.

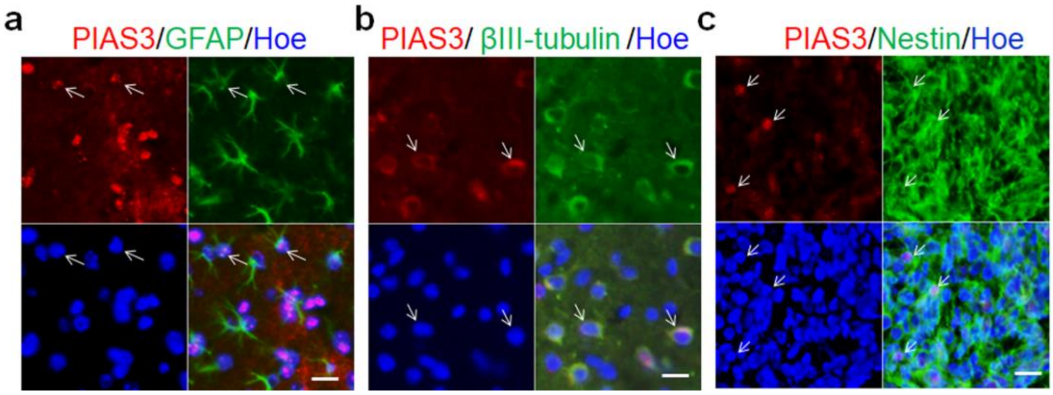

Supplementary Figure 4. Sub-cellular localization of PIAS3 in normal human brain and GBM tissue. **(a)** Double IF staining for GFAP and PIAS3 of normal human brain section. **(b)** Double IF staining for βIII-tubulin and PIAS3 of normal human brain section. **(c)** Double IF staining for Nestin and PIAS3 of GBM tissue section. Arrows indicated the representative PIAS3 positive cells. Scale bars, 20 μm.

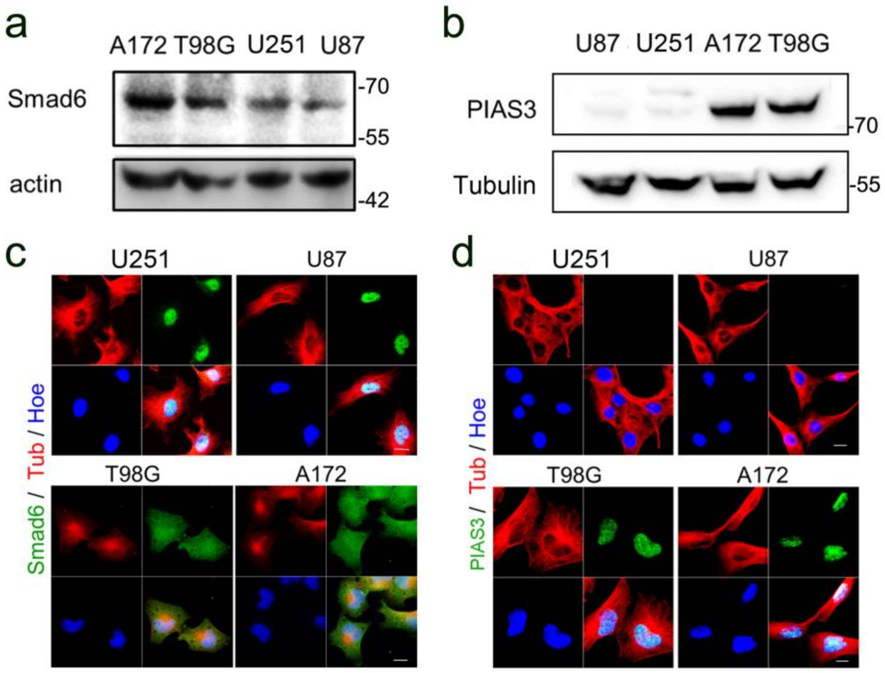

Supplementary Figure 5. Sub-cellular localization of Smad6 and PIAS3 protein expression in GBM cell lines. **(a)** Smad6 protein expression in GBM cell lines. **(b)** Double IF labeling of Smad6 (red) and β-tubulin (Tubulin, green) showed different sub-cellular localization of Smad6 in GBM cell lines. Hoechst (blue) stained cell nuclei. **(c)** PIAS3 protein expression in GBM cell lines. **(d)** Double IF staining for PIAS3 and β-tubulin in GBM cell lines. Scale bars, 20 μm.

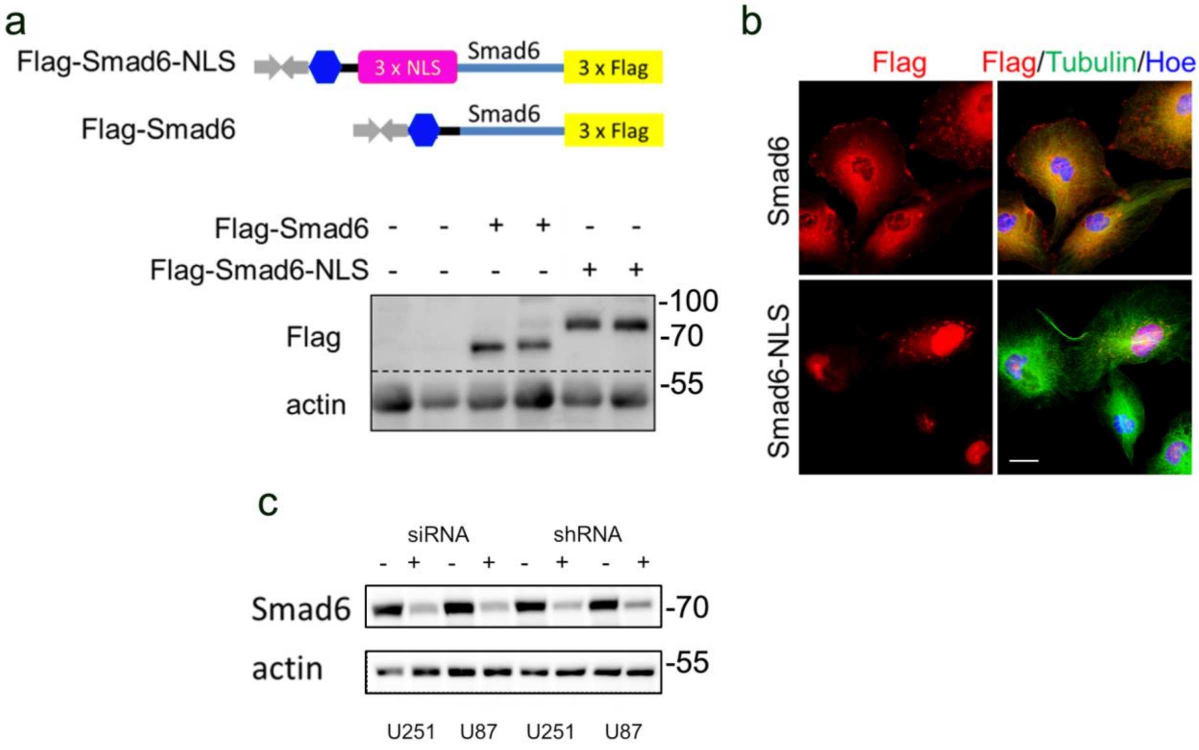

Supplementary Figure 6. Smad6 overexpression and knock-down constructs. **(a)** Schematic representation of forced nuclear Smad6 expression construct (upper panel) and Western blot analysis (lower panel). Flag-Smad6-NLS encodes Smad6 with three copies of the nuclear localization signal (NLS) fused at its N-terminus and 3 x Flag tag fused at its C-terminus. **(b)** Double IF staining for Flag (red) and β-tubulin (green) showed non-nuclear localization of Flag-Smad6 and nuclear localization of Flag-Smad6-NLS in T98G cells. Scale bars, 20 μm**.** **(c)** Western blot analysis of Smad6 expression in U251 and U87 cells with siRNA or shRNA mediated Smad6 knock-down. GAPDH served as a loading control.

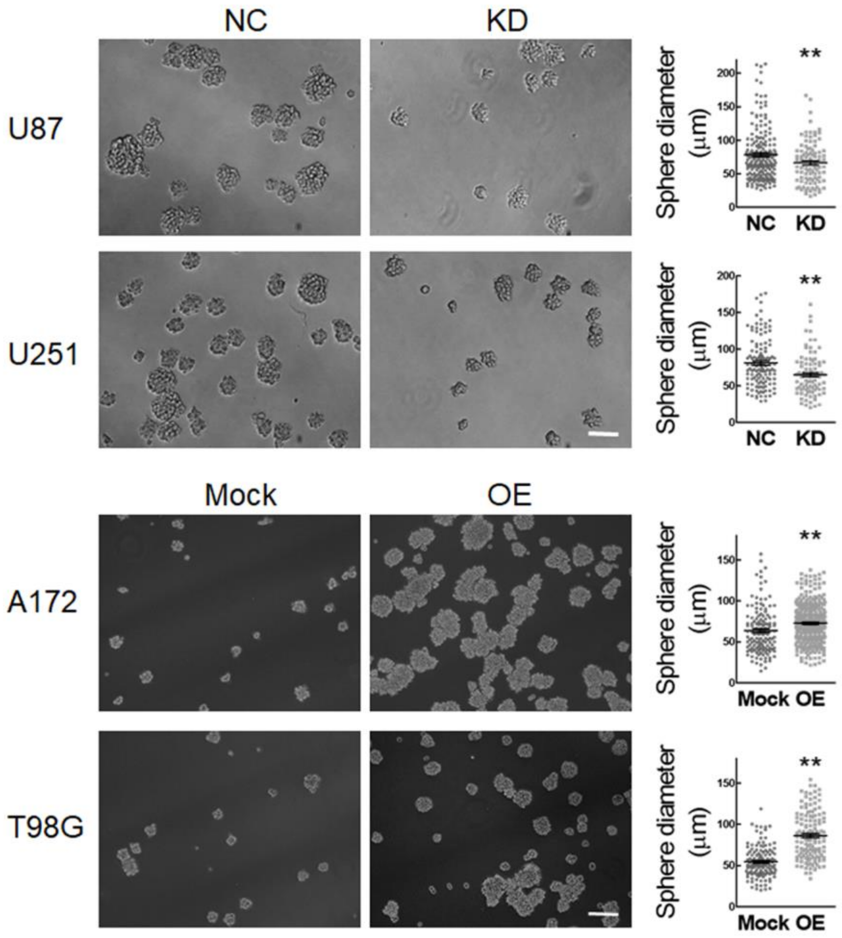

Supplementary Figure 7. Nuclear-Smad6 promotes secondary tumor sphere formation of GBM cells. Representative images of in vitro secondary tumor sphere formation in U87 and U251 cells with lentivirus mediated Smad6 knock-down (KD; upper panel) and T98G and A172 cells with adenovirus mediated nuclear-Smad6 OE (upper panel). Nuclear-Smad6 OE promoted tumor sphere formation, whereas Smad6 KD inhibited secondary sphere formation of glioblastoma cells . Scale bars, 200 μm. Data were represented as means ± s.d. and analyzed using Unpaired Student's *t* test. $^{**}P<0.01$.

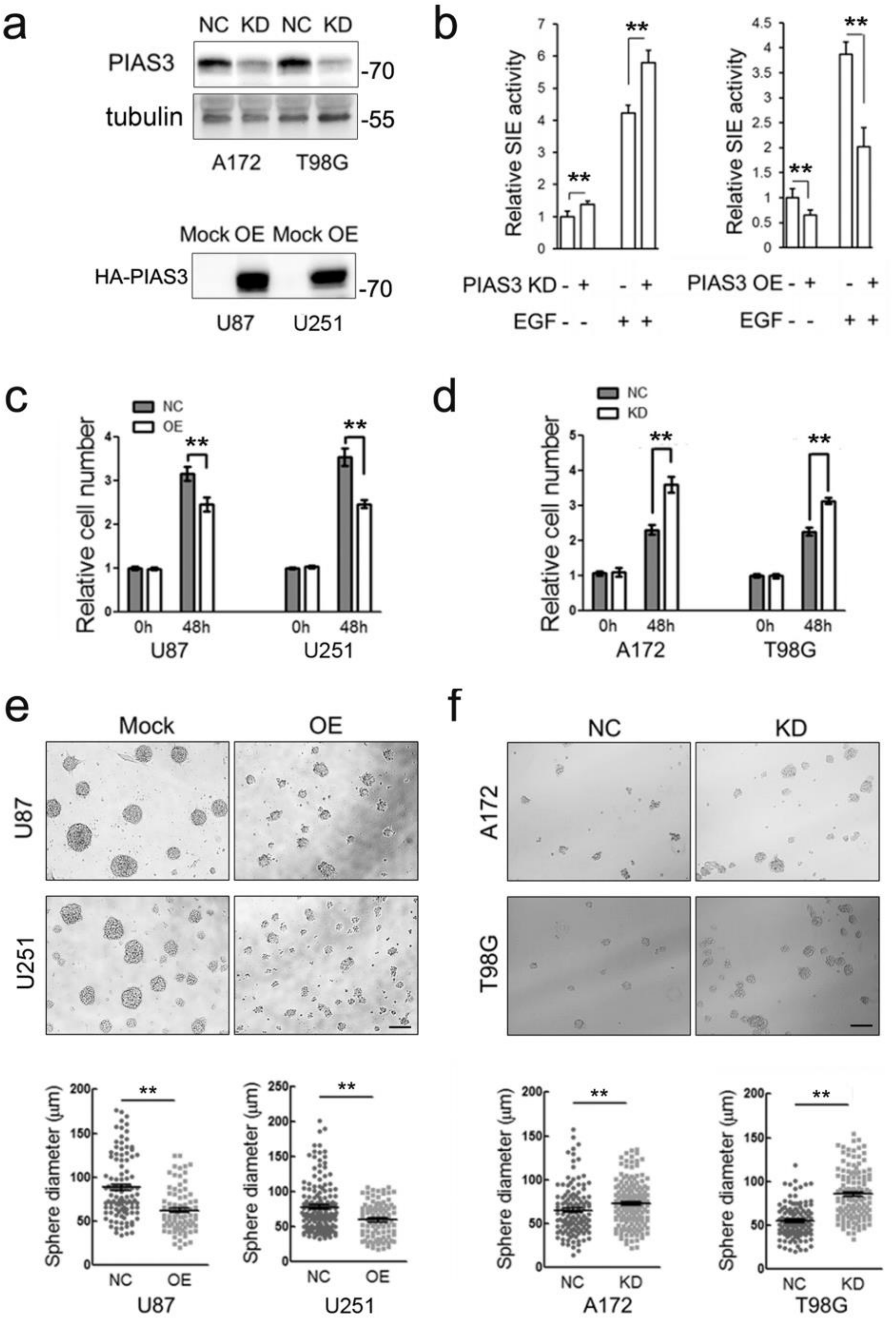

Supplementary Figure 8. PIAS3 negatively regulates glioma cell growth. **(a)** Western blot analysis of PIAS3 expression in indicated cell lines after PIAS3 KD (left panel) and overexpression (right panel). **(b)** PIAS3 inhibited STAT3 transcriptional activity. T98G cells were transfected with PIAS3 siRNA (left panel) and U87 cells were transfected with HA-PIAS3 (right panel) followed by transfection of SIE and pRL-TK vectors. EGF (60 ng ml^{-1}) was added 6 h before assay. Data represent means ± s.d. (***P*<0.01, Student's *t* test. *n*=3). **(c)** Overexpression of PIAS3 inhibited cell growth of U87 and U251 cells (***P*<0.01, Student's *t* test, bars indicate means ±s.d. *n*=6). **(d)** PIAS3 KD promoted cell growth of A172

and T98G cells (**P<0.01, Student's t-test, bars indicate means ± s.d. n=6). **(e)** PIAS3 overexpression inhibited tumor sphere formation of U87 and U251 cells (**P<0.001, Unpaired t test). Tumor spheres were derived from stable PIAS3 overexpression (OE) or empty plasmid (Mock) cells to measure the tumor sphere diameter. Scale bars, 200 μm. **(f)** PIAS3 knock-down promoted tumor sphere formation of A172 and T98G cells (**P<0.001, Unpaired t test). Scale bars, 200 μm.

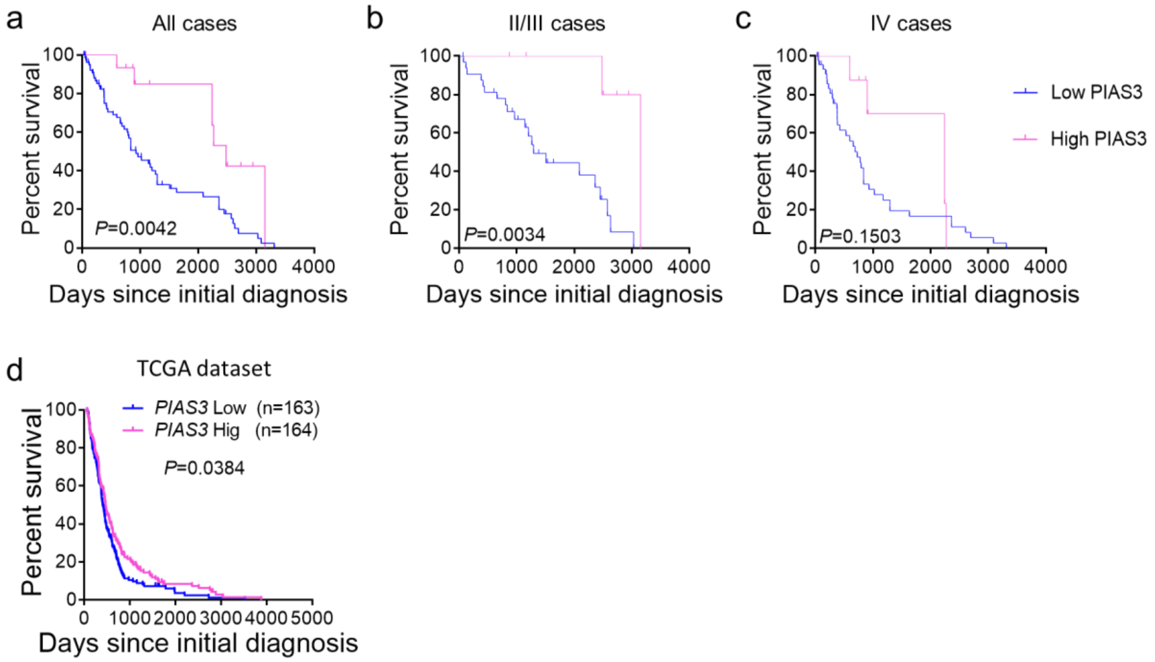

Supplementary Figure 9. Low PIAS3 expression in gliomas predicts poor survival. **(a-c)** Survival analysis of patients from IHC array based on all cases and different grades of gliomas. **(d)** Survival analysis according to the *PIAS3* mRNA expression in TCGA GBM dataset (Log-rank χ^2=4.288, P=0.0384).

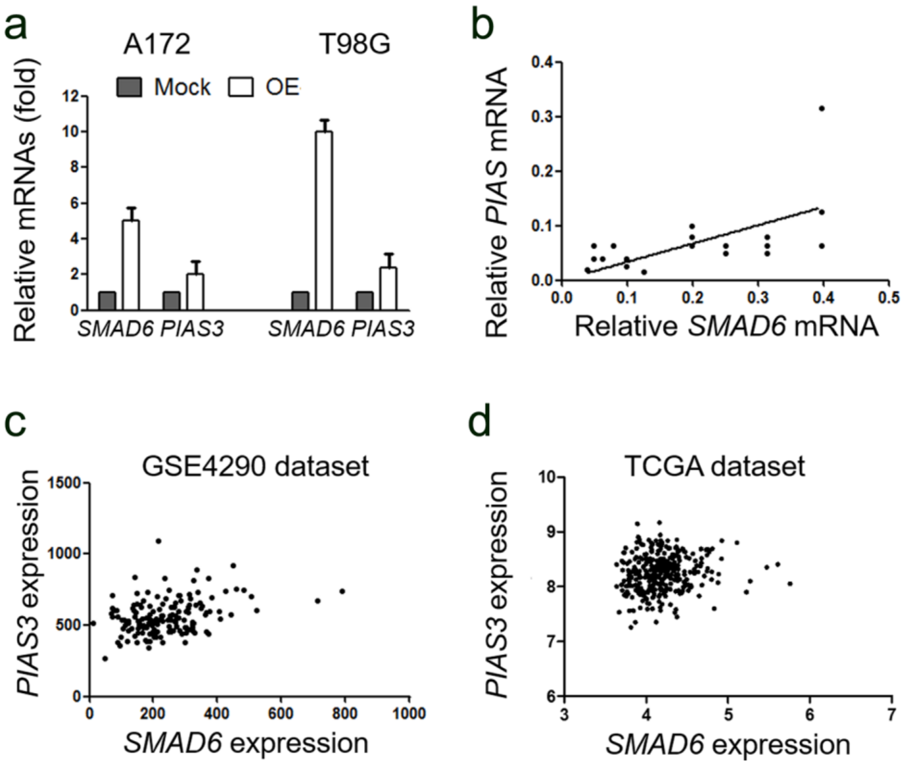

Supplementary Figure 10. *SMAD6* positively correlates to *PIAS3* in gliomas at mRNA level. **(a)** Overexpression of nuclear-Smad6 increased *PIAS3* mRNA (*P*<0.001, Student's t-test, bars indicate means ± s.d. *n*=3). A172 and T98G cells transfected with control or nuclear-Smad6 expression plasmids were subjected to RT-qPCR analysis for related gene expression to the endogenous control gene, *GAPDH*. **(b)** RT-qPCR showed a positive correlation between *SMAD6* and *PIAS3* in primary glioma tissues (Spearman *r*=0.6359, *P*=0.0026. *n*=20). **(c)** The GSE4290 dataset showed that *PIAS3* positively correlated with *SMAD6* expression at the mRNA level in gliomas (GDS1962/209886_s_at/*SMAD6*; 203035_s_at/ *PIAS3*; Spearman *r*=0.299, *P*<0.001, *n*=77). **(d)** TCGA dataset showed that *PIAS3* positively correlated with *SMAD6* mRNA in gliomas (Spearman *r*=0.141, *P*<0.0106. *n*=327).

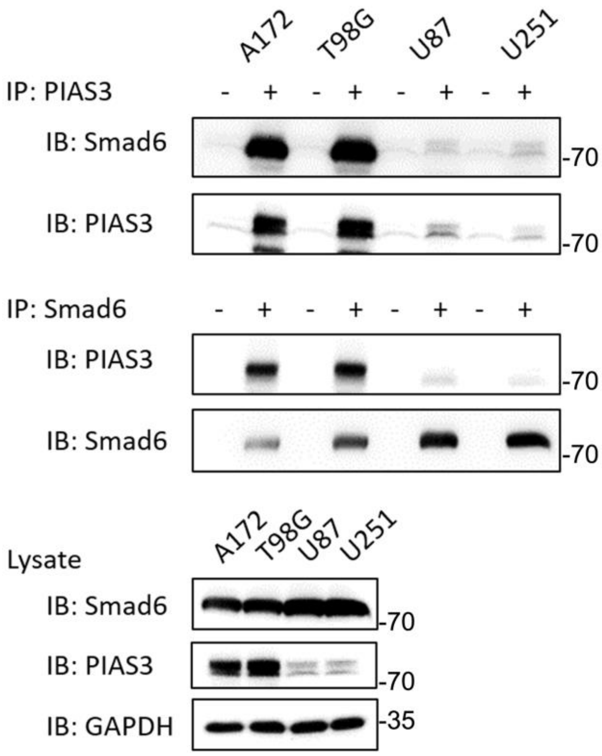

Supplementary Figure 11. Smad6 and PIAS3 interact *in vivo* in glioma cells. IP experiments were performed in proteins from indicated cell lines. Rabbit IgG antibody was used as a negative control.

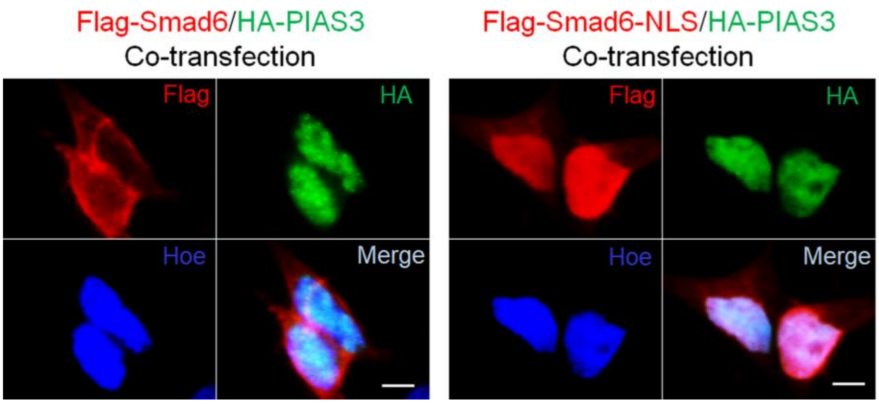

Supplementary Figure 12. Smad6 and PIAS3 expression constructs shows different cellular localization. Double immunofluorescence staining for Flag (red) and HA (green) showed non-nuclear expression of Flag-Smad6 and nuclear expression of Flag-Smad6-NLS in 293T cells after transfection. Hoechst (blue) stained the cell nuclei. Scale bars, 20 μm.

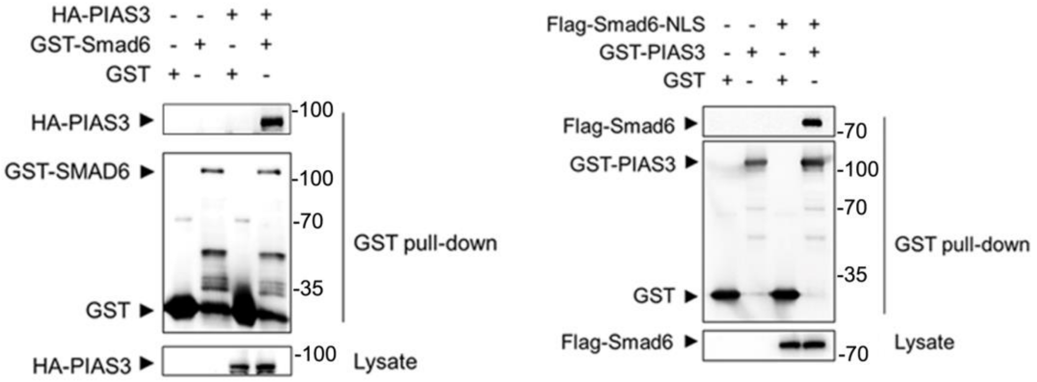

Supplementary Figure 13. Smad6 interacts to PIAS3. A GST pull-down assay was performed to detect Smad6-PIAS3 interaction. 293T cells were transfected with HA-PIAS3 (left panel) or Flag-Smad6-NLS (right panel).

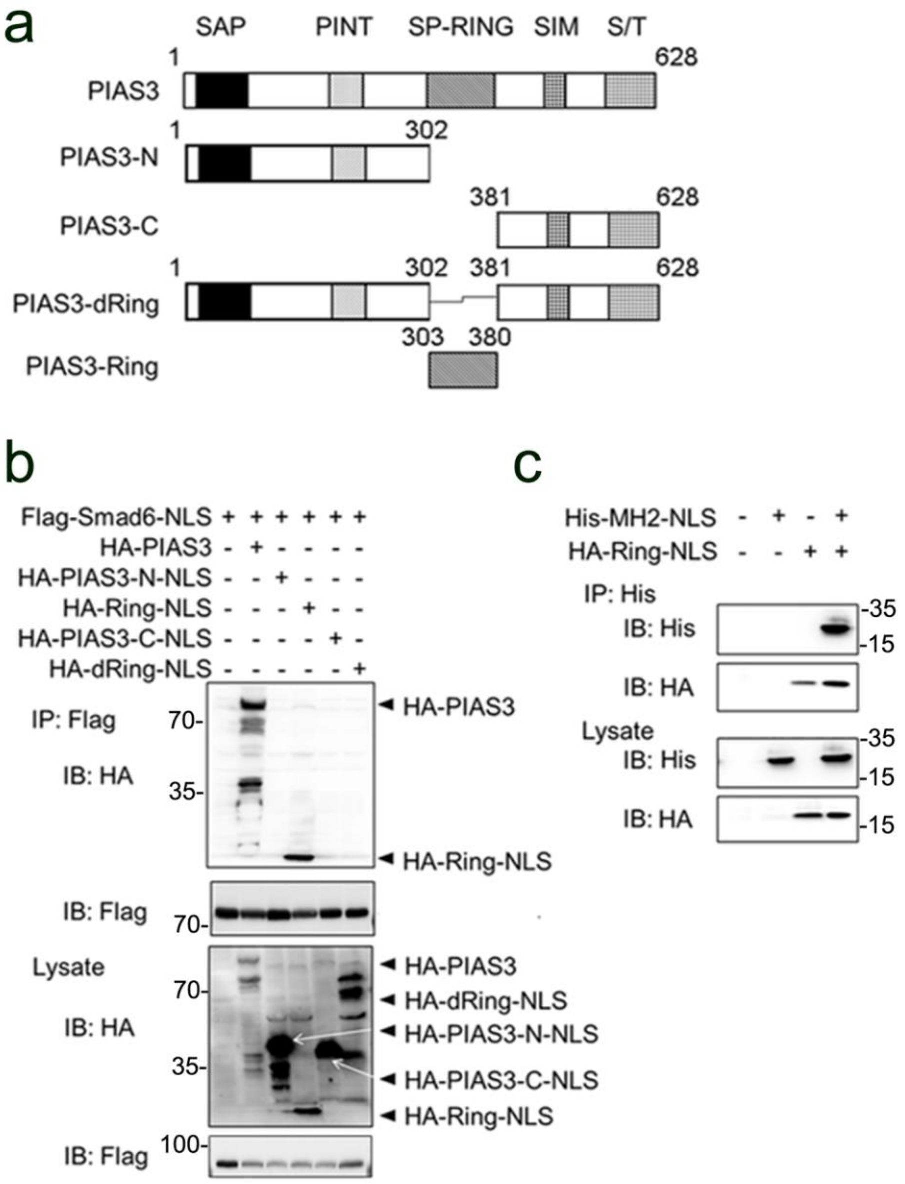

Supplementary Figure 14. Ring domain is essential for the interaction of PIAS3 with Smad6. **(a)** Schematic diagram of PIAS3 and its deletion mutants. **(b)** Mapping of the domain of PIAS3 for its interaction with Smad6. 293T cells were co-transfected with HA-Smad6-NLS and HA-tagged nuclear PIAS3 deletion mutants, followed by IP with anti-Flag antibody and IB analysis with indicated antibodies. **(c)** PIAS3 Ring domain bound to Smad6 MH2 domain. 293T cells were co-transfected with HA-Ring-NLS and His-MH2-NLS and followed by IP with anti-His antibody.

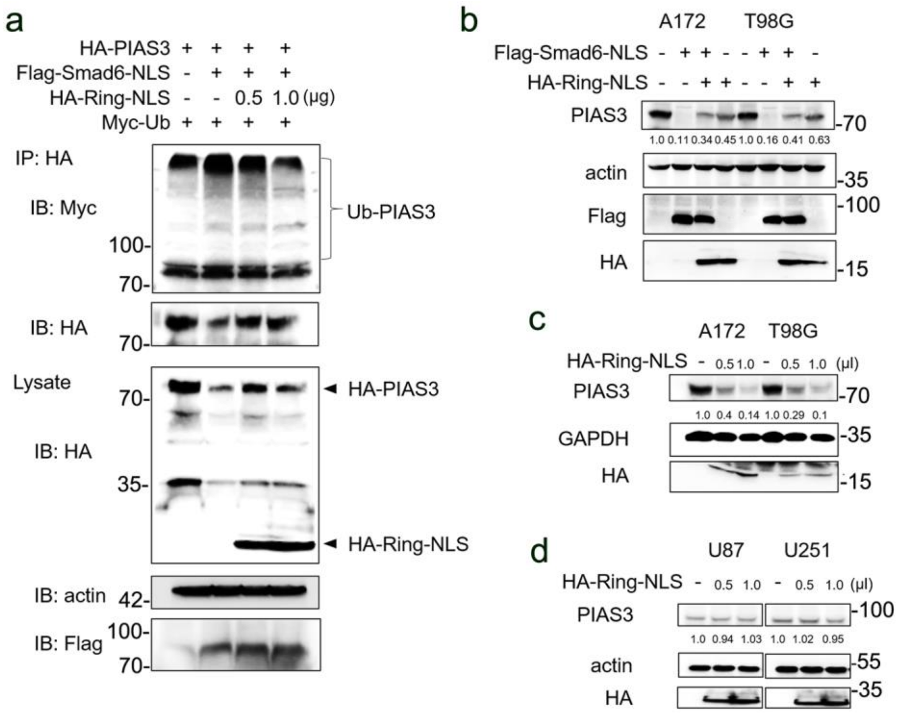

Supplementary Figure 15. Nuclear-Ring antagonizes PIAS3 ubiquitination and degradation induced by nuclear-Smad6. **(a)** Nuclear-Ring antagonizes PIAS3 ubiquitination and degradation induced by nuclear-Smad6 in a dose dependent manner. **(b)** Nuclear-Ring construct antagonizes endogenous PIAS3 downregulation induced by nuclear-Smad6 overexpression. A172 or T98G cells were infected with nuclear-Smad6 or nuclear-Ring adenovirus construct, followed by WB with indicated antibodies. The semi-quantification of PIAS3 was listed. **(c)** Nuclear-Ring construct induced endogenous PIAS3 downregulation. A172 or T98G cells were infected nuclear-Ring adenovirus construct, followed by WB with indicated antibodies. **(d)** Nuclear-Ring construct had no effect on endogenous PIAS3 expression in U87 and

U251 cells. U87 and U251 cells were infected nuclear-Ring adenovirus construct, followed by WB with indicated antibodies.

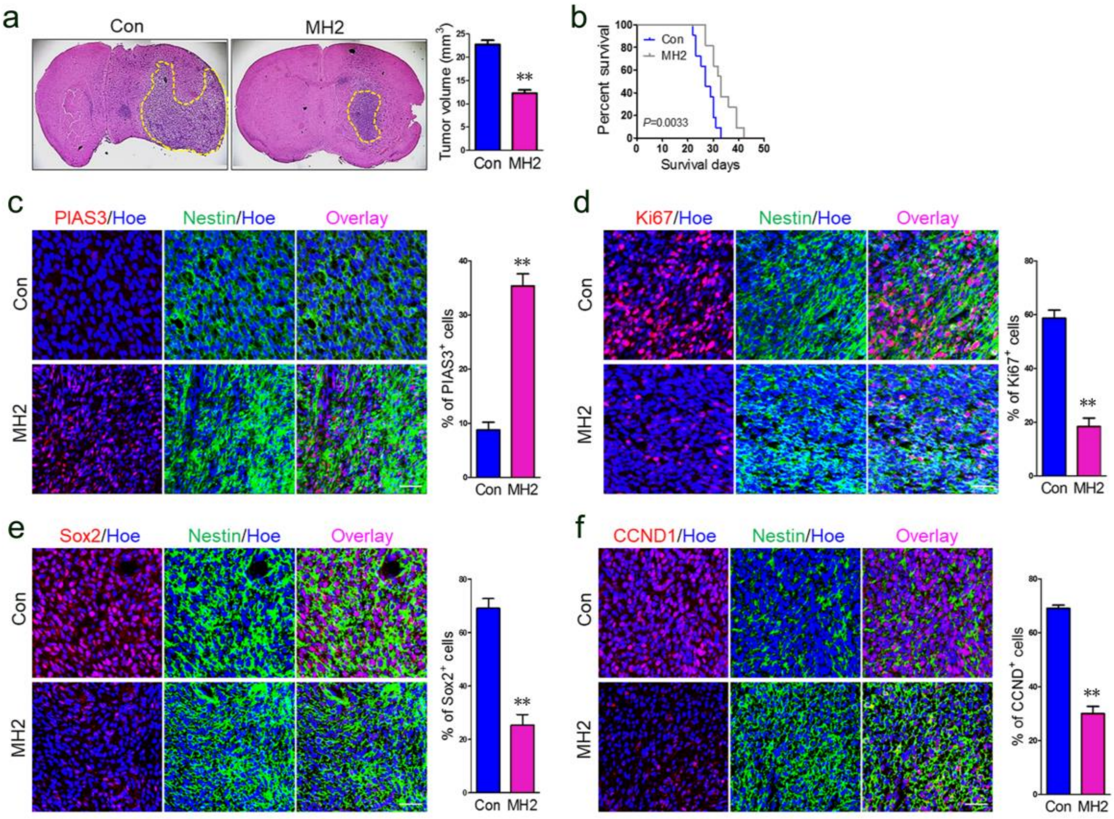

Supplementary Figure 16 TAT-MH2-NLS potently inhibits patient-derived glioma cell tumor growth and prolongs the animal survival. **(a)** TAT-MH2-NLS inhibits patient-derived glioma cell tumor growth (*n*=5). Representative images of tumor formed by patient-derived T06 cells in the mouse brain (left panel) and quantification (right panel) of treated xenografts. Xenografts were collected at 20 days after tumor implantation. Yellow lines showed the tumors. **(b)** TAT-MH2-NLS treatment prolongs the animal survival. Kaplan-Meier survival curves of mice implanted with T06 cells (Log-rank χ^2=8.618, *P*=0.0033. *n*=10). **(c-f)** Representative double IF images (left panel) and the quantification of (right panel) of indicated proteins expression in xenografts derived from T06 cells (*n*=5). Scale bars, 25 μm. Data were represented as means ± s.d. in (a, c-f) and analyzed using two-tailed Student's *t* test. ***P*<0.01.

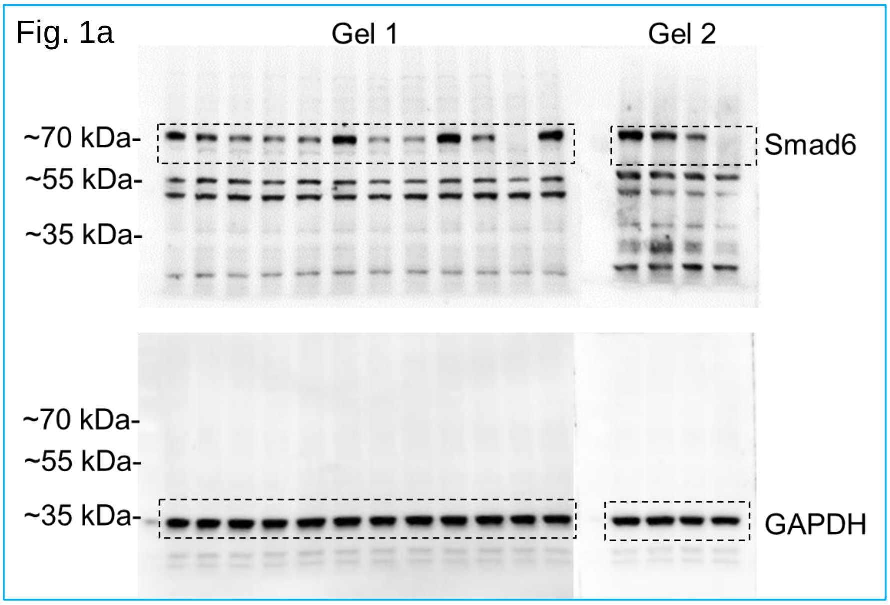

Fig. 1a
Gel 1
Gel 2
~70 kDa-
~55 kDa-
~35 kDa-
Smad6
~70 kDa-
~55 kDa-
~35 kDa-
GAPDH

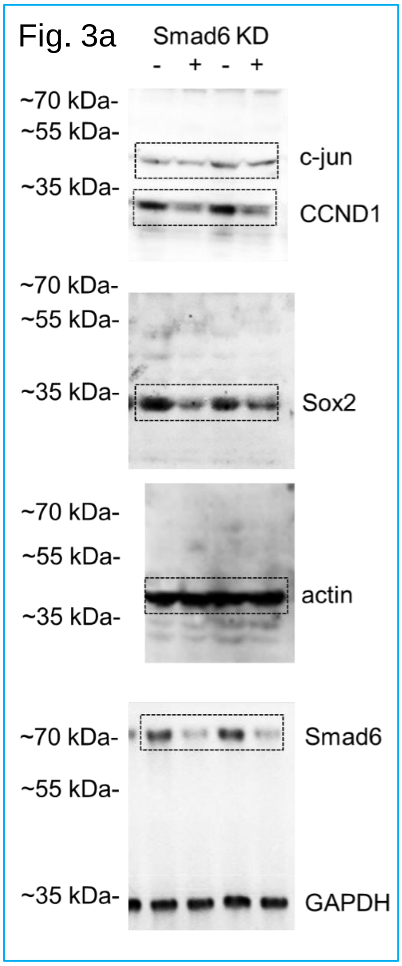

Fig. 3a
Smad6 KD
- + - +
~70 kDa-
~55 kDa-
~35 kDa-
c-jun
CCND1
~70 kDa-
~55 kDa-
~35 kDa-
Sox2
~70 kDa-
~55 kDa-
~35 kDa-
actin
~70 kDa-
~55 kDa-
~35 kDa-
Smad6
GAPDH

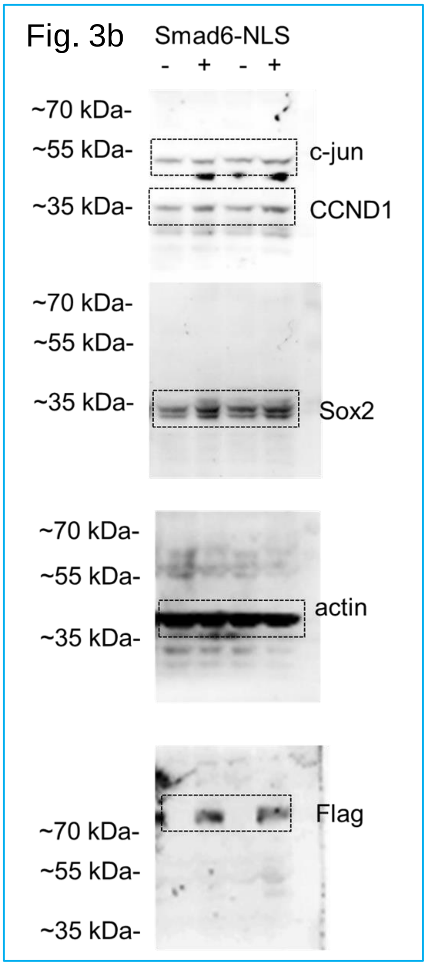

Fig. 3b
Smad6-NLS
- + - +
~70 kDa-
~55 kDa-
~35 kDa-
c-jun
CCND1
~70 kDa-
~55 kDa-
~35 kDa-
Sox2
~70 kDa-
~55 kDa-
~35 kDa-
actin
Flag
~70 kDa-
~55 kDa-
~35 kDa-

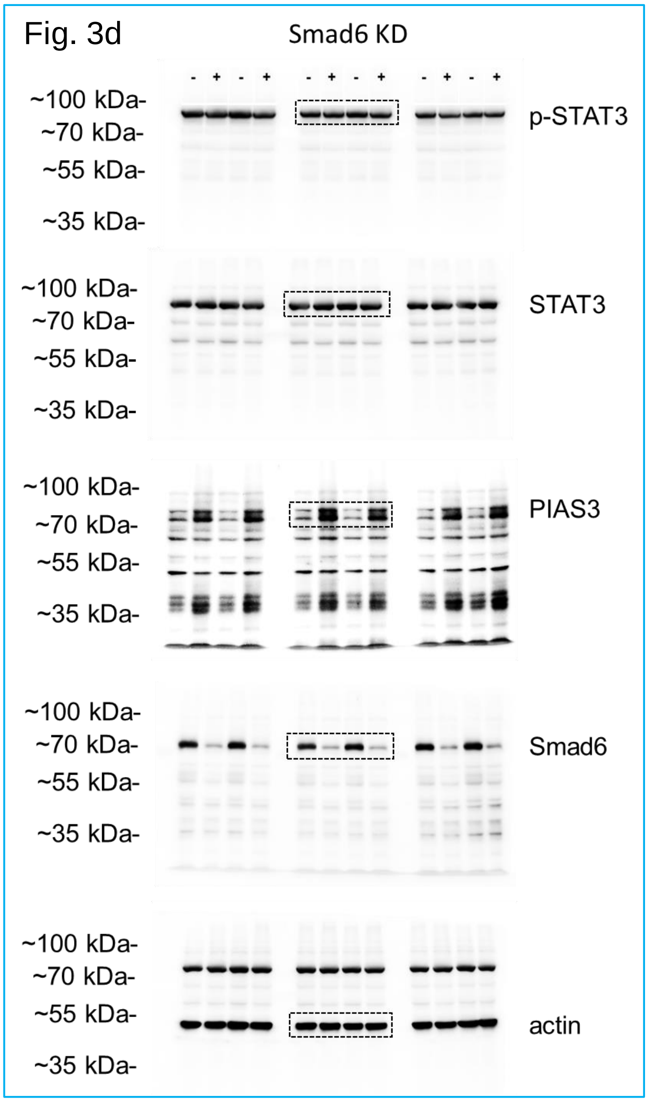
Fig. 3d
Smad6 KD
- + - + - + - + - + - +
~100 kDa-
~70 kDa-
~55 kDa-
~35 kDa-
p-STAT3
STAT3
PIAS3
Smad6
actin

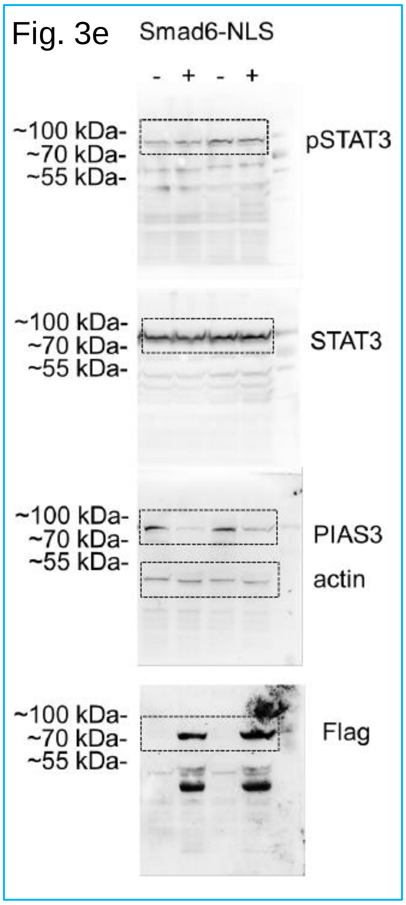
Fig. 3e
Smad6-NLS
- + - +
~100 kDa-
~70 kDa-
~55 kDa-
pSTAT3
STAT3
PIAS3
actin
Flag

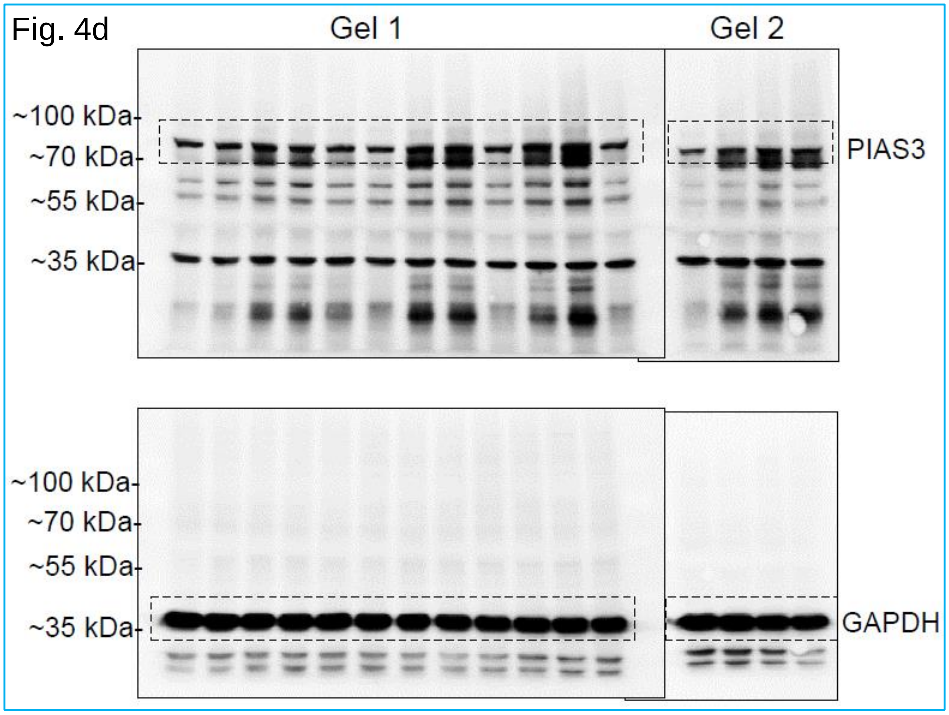
Fig. 4d
Gel 1
Gel 2
~100 kDa
~70 kDa
~55 kDa
~35 kDa
PIAS3
GAPDH

Fig. 5a

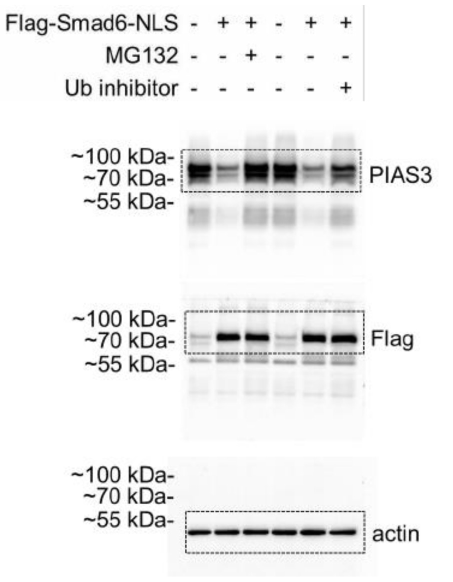

Fig. 5b

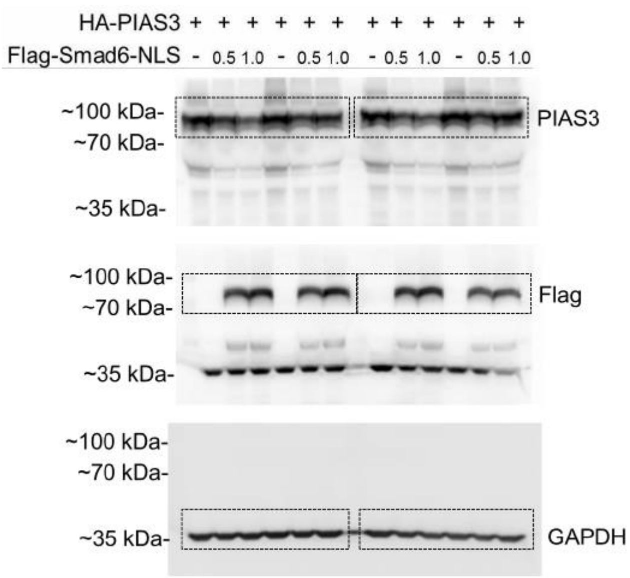

Fig. 5c

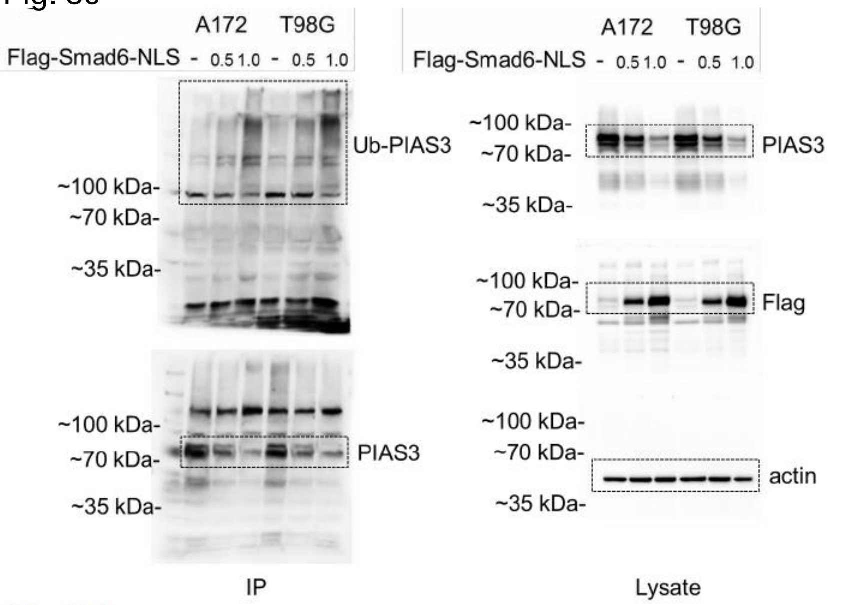

Fig. 5d

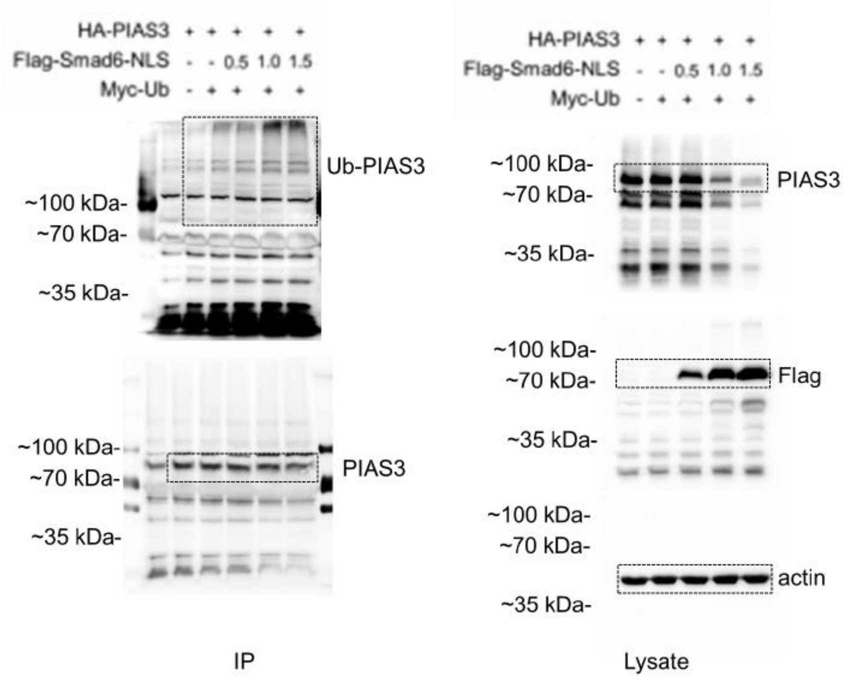

Fig. 6a

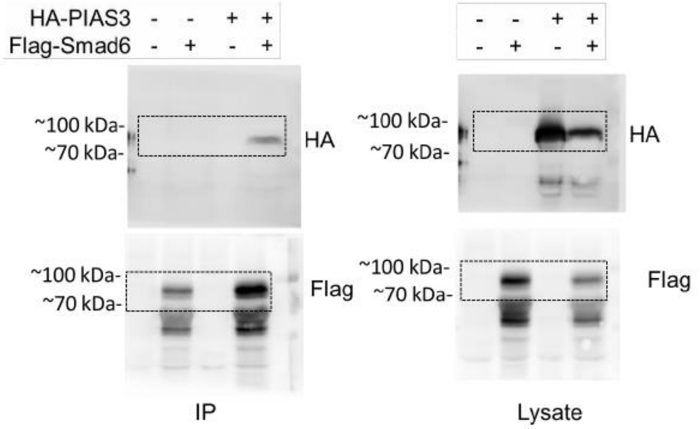

Fig. 6b

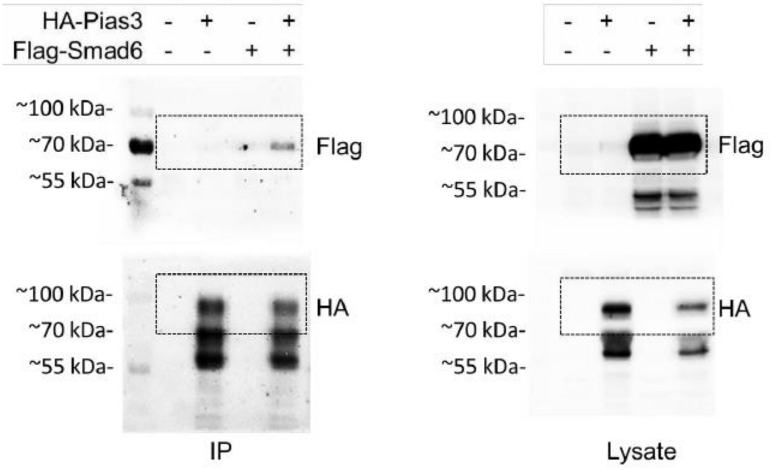

Fig. 6d

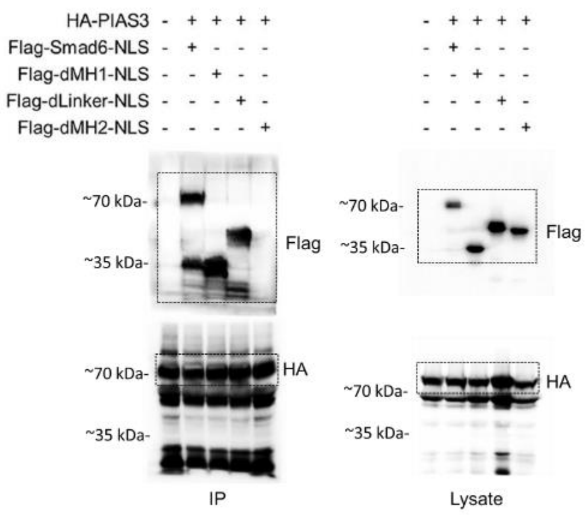

Fig. 6e

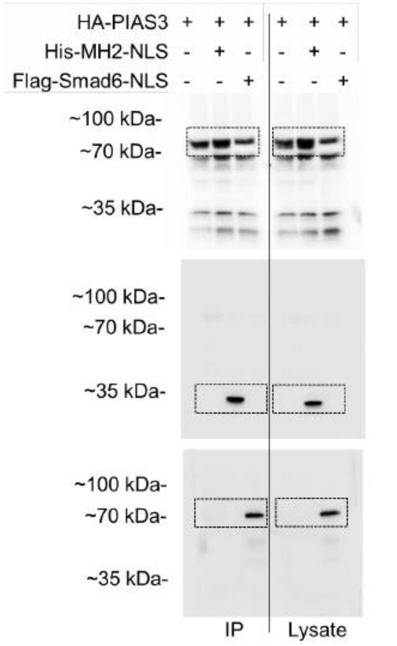

Fig. 7a

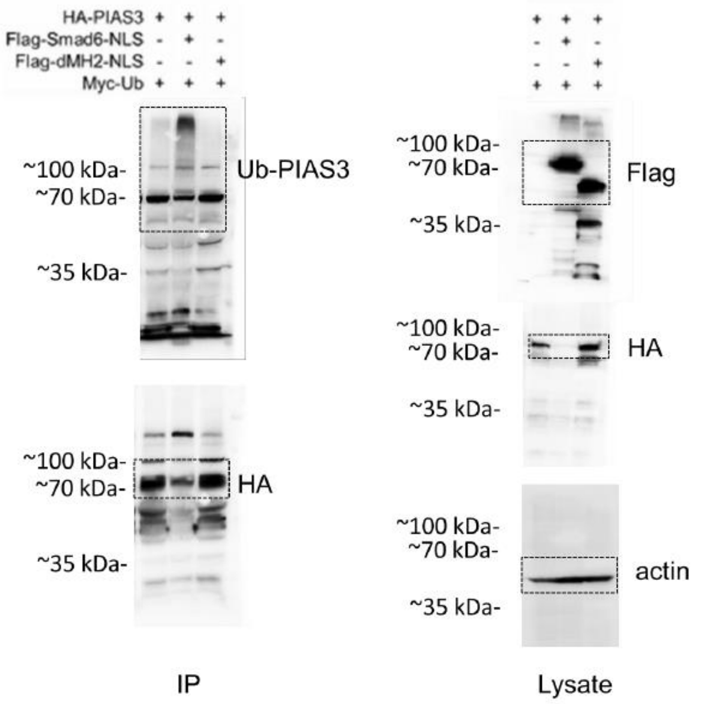

Fig. 7b

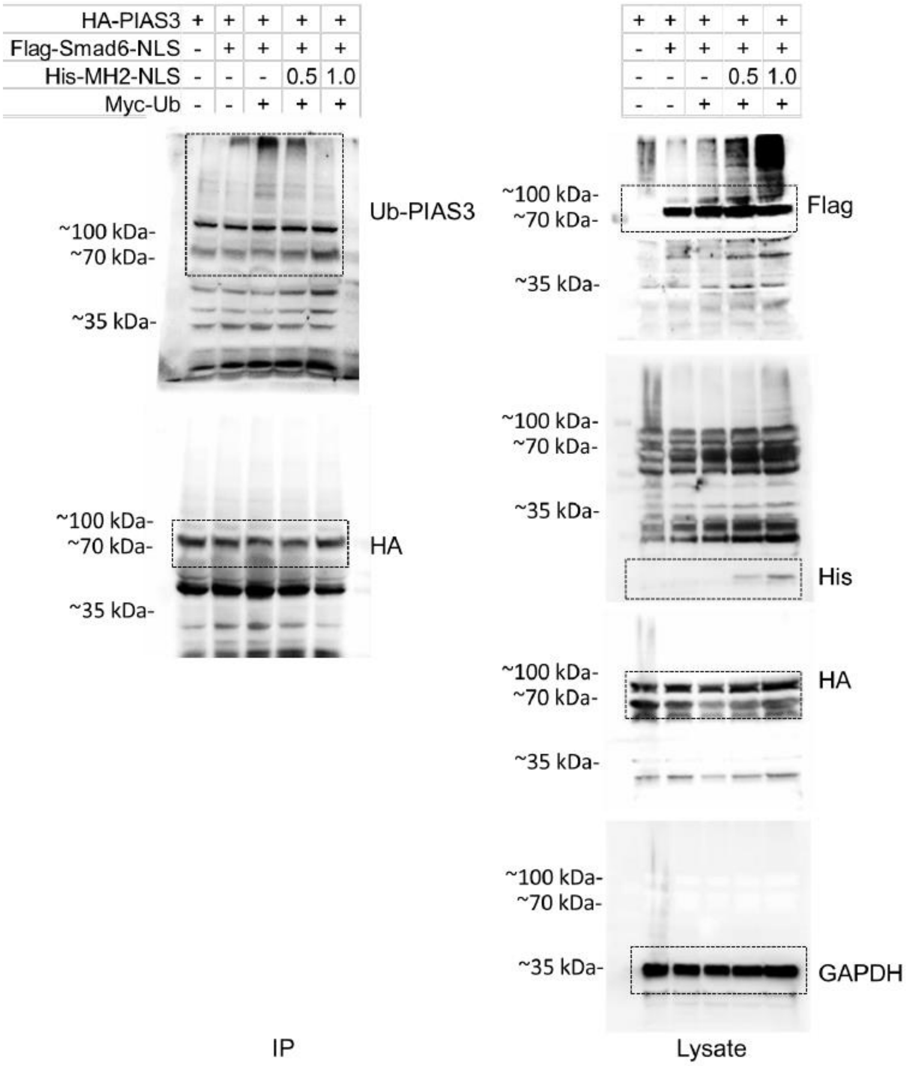
HA-PIAS3 + + + + +
Flag-Smad6-NLS - + + + +
His-MH2-NLS - - - 0.5 1.0
Myc-Ub - - + + +
Ub-PIAS3
HA
Flag
His
HA
GAPDH
~100 kDa-
~70 kDa-
~35 kDa-
IP
Lysate

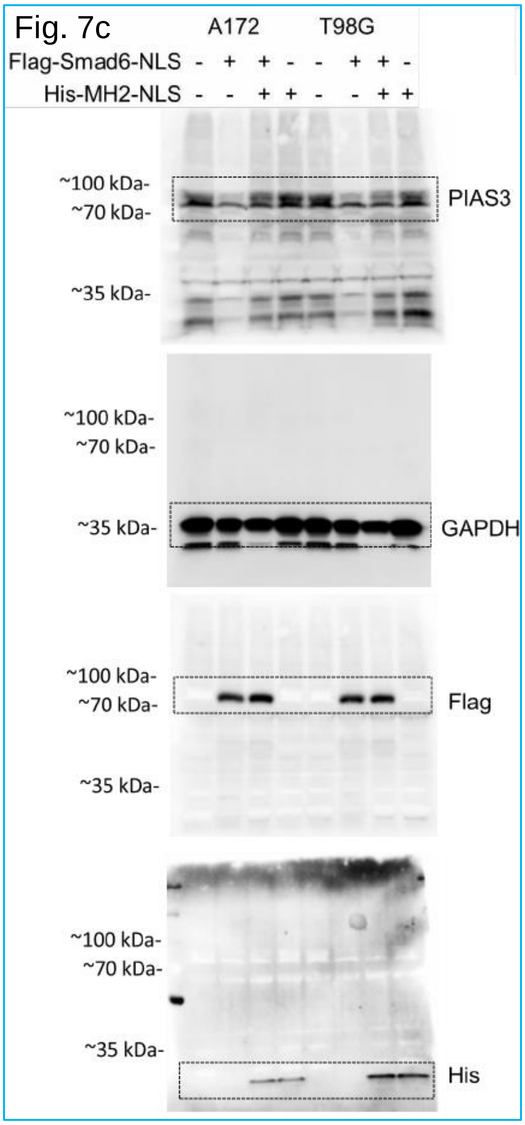

Fig. 7c
A172
T98G
Flag-Smad6-NLS - + + - - + + -
His-MH2-NLS - - + + - - + +
~100 kDa-
~70 kDa-
~35 kDa-
PIAS3
~100 kDa-
~70 kDa-
~35 kDa-
GAPDH
~100 kDa-
~70 kDa-
~35 kDa-
Flag
~100 kDa-
~70 kDa-
~35 kDa-
His

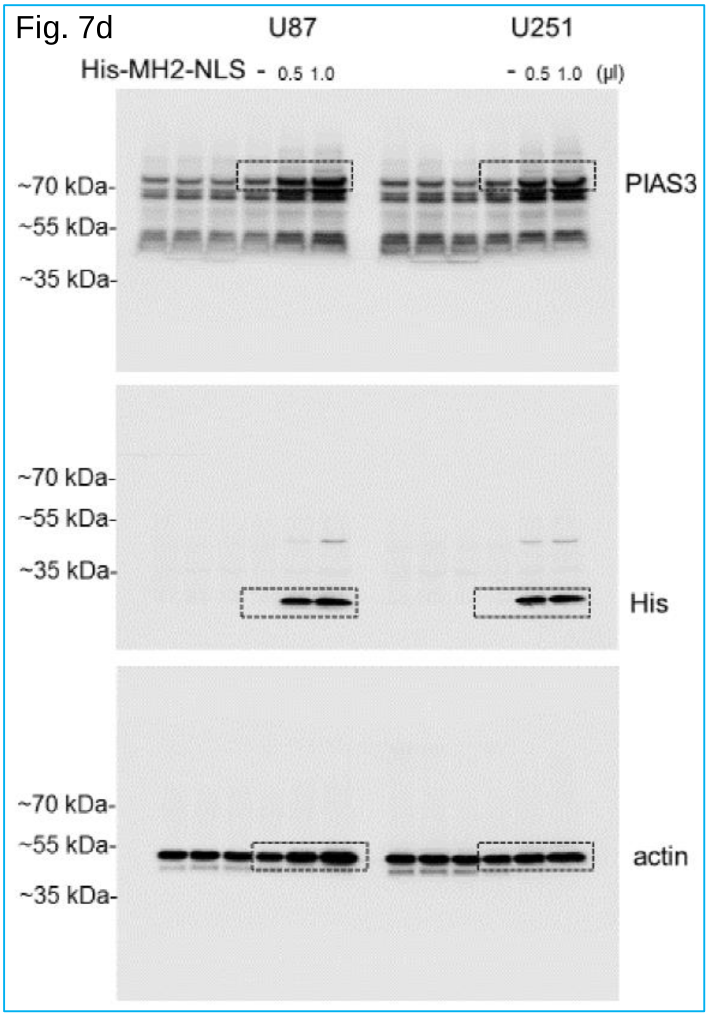

Fig. 7d
U87
U251
His-MH2-NLS - 0.5 1.0
- 0.5 1.0 (µl)
~70 kDa-
~55 kDa-
~35 kDa-
PIAS3
~70 kDa-
~55 kDa-
~35 kDa-
His
~70 kDa-
~55 kDa-
~35 kDa-
actin

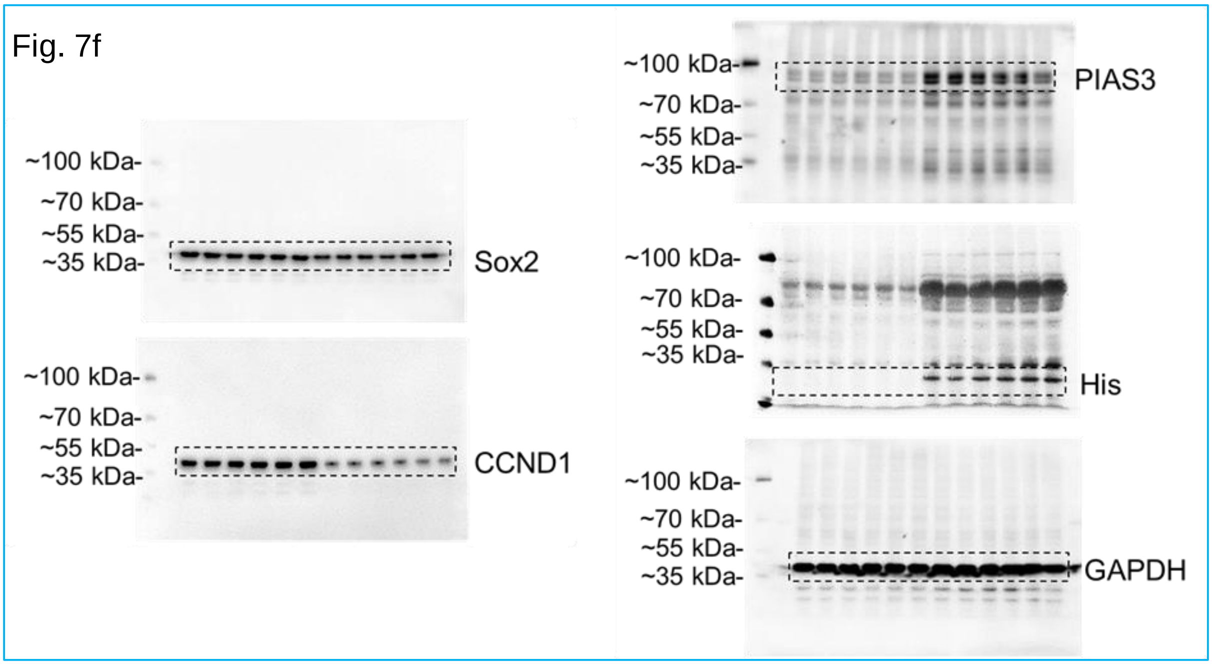

Fig. 7f
~100 kDa-
~70 kDa-
~55 kDa-
~35 kDa-
Sox2
~100 kDa-
~70 kDa-
~55 kDa-
~35 kDa-
CCND1
~100 kDa-
~70 kDa-
~55 kDa-
~35 kDa-
PIAS3
~100 kDa-
~70 kDa-
~55 kDa-
~35 kDa-
His
~100 kDa-
~70 kDa-
~55 kDa-
~35 kDa-
GAPDH

Fig. 8a

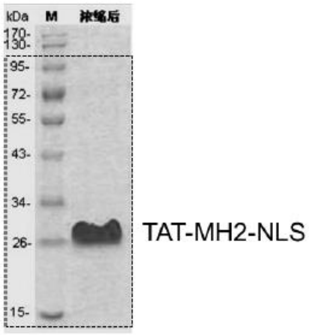

Fig. 8c

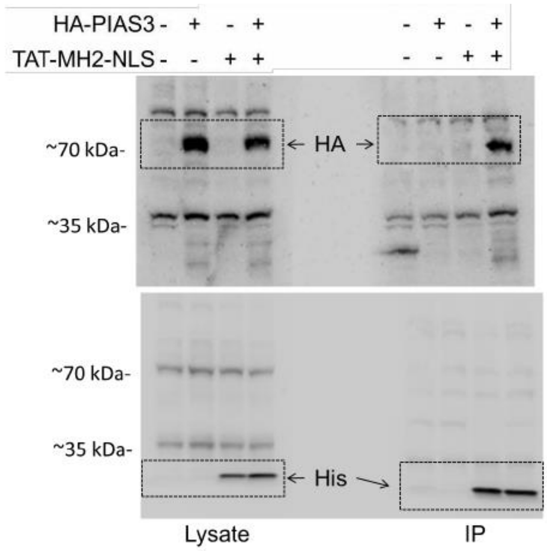

Fig. 8d

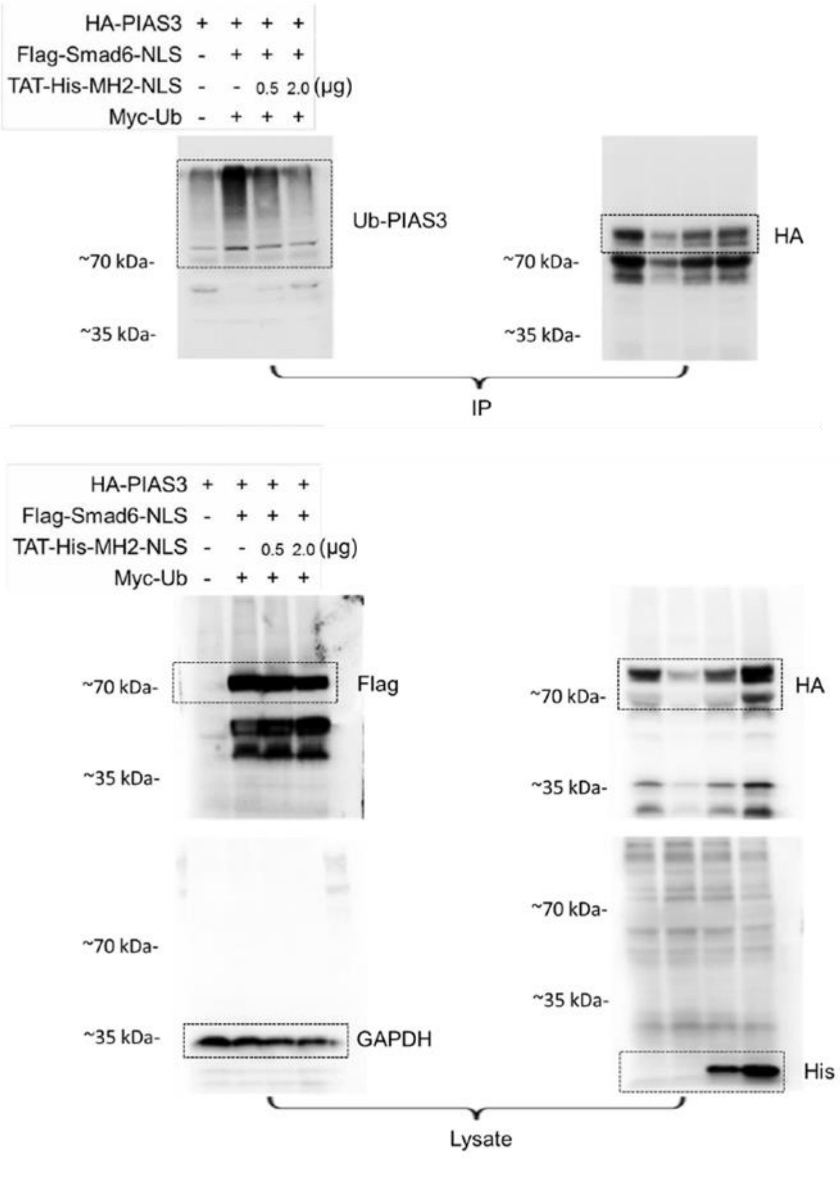

Fig. 8f

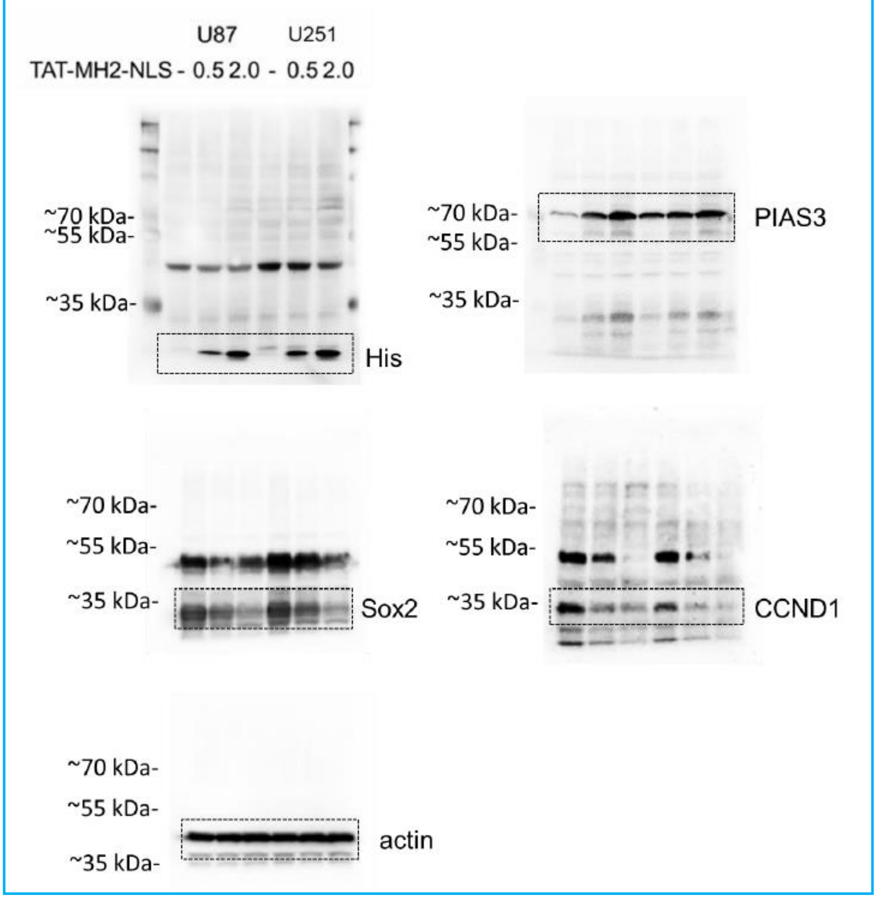

Fig. 9a

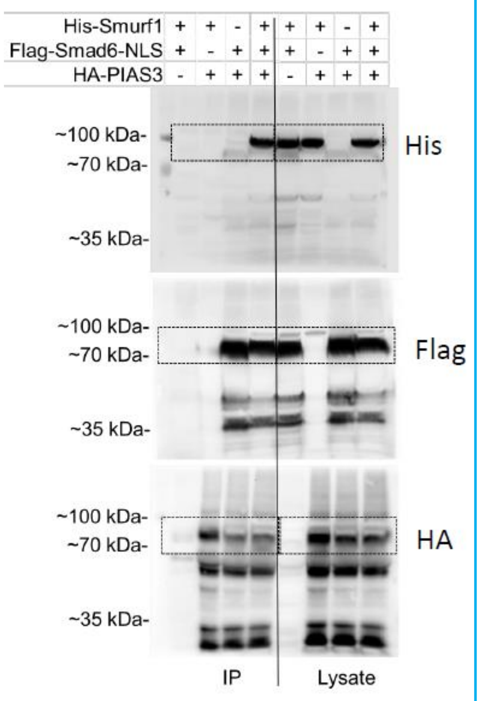

Fig. 9b

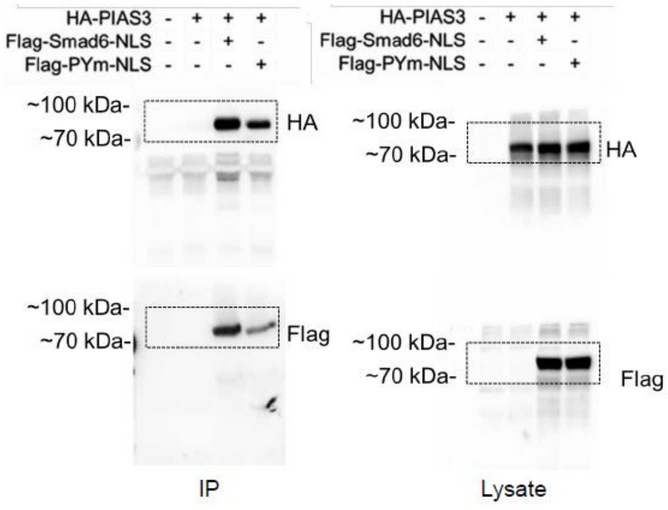

Fig. 9c

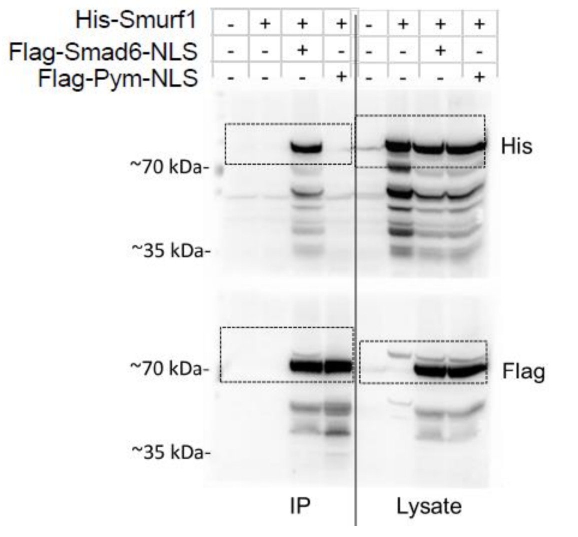

His-Smurf1
Flag-Smad6-NLS
Flag-Pym-NLS
~70 kDa-
~35 kDa-
His
Flag
IP
Lysate

Fig. 9d

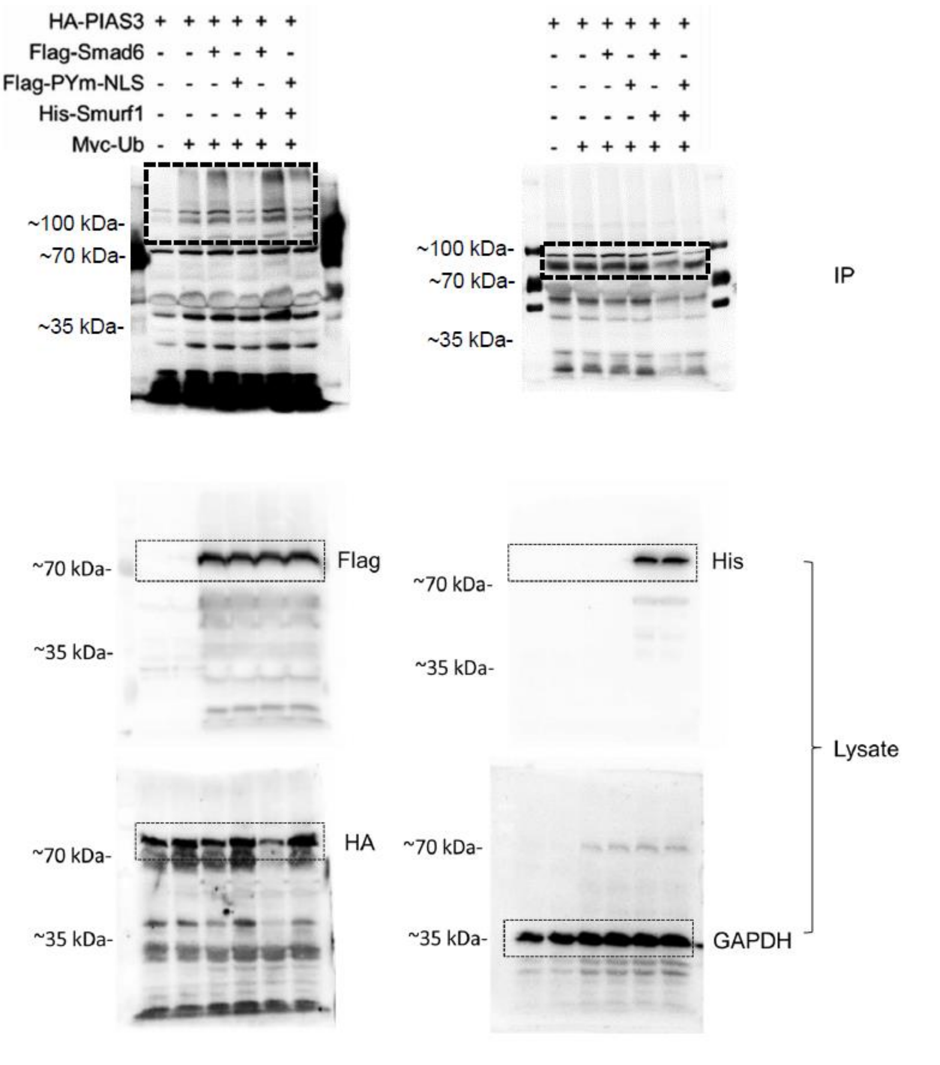

HA-PIAS3
Flag-Smad6
Flag-PYm-NLS
His-Smurf1
Myc-Ub
~100 kDa-
~70 kDa-
~35 kDa-
IP
Flag
His
HA
GAPDH
Lysate

Fig. 9e

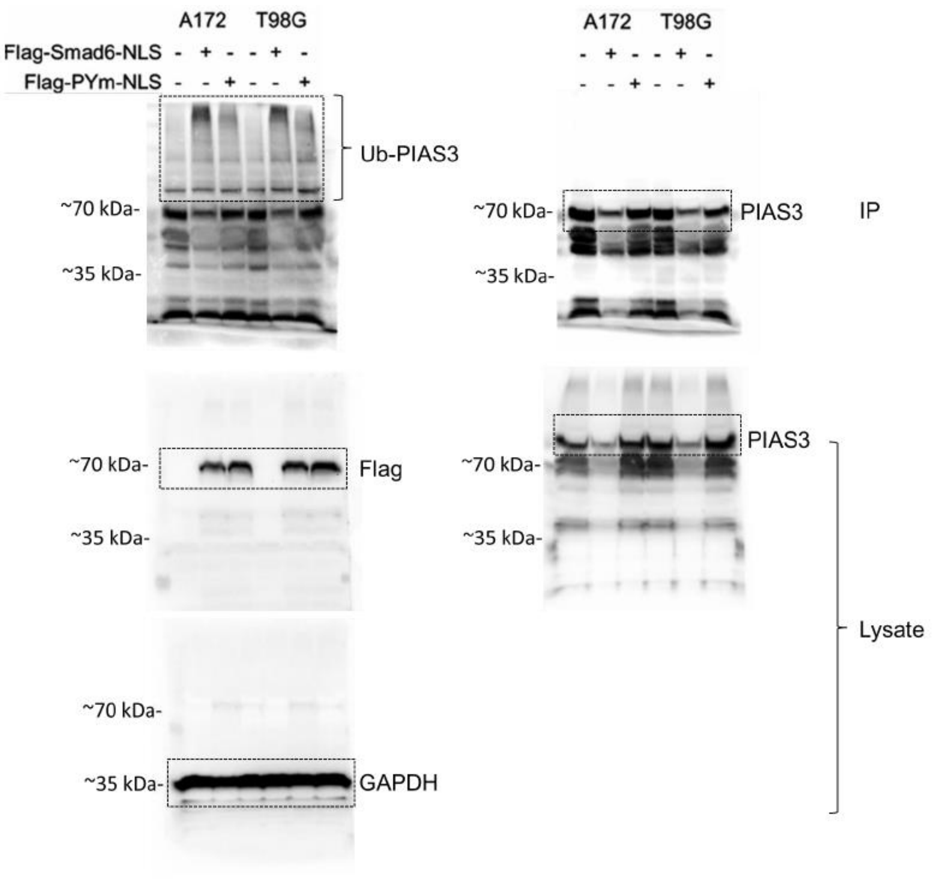

S Fig. 5a

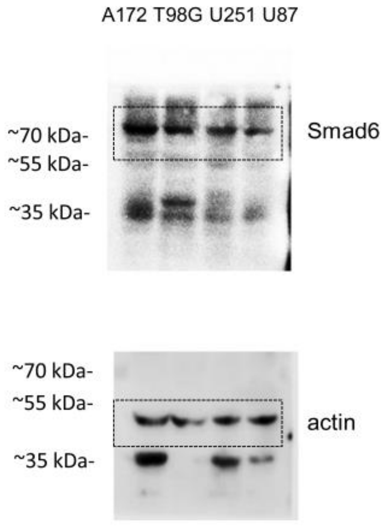

S Fig. 5c

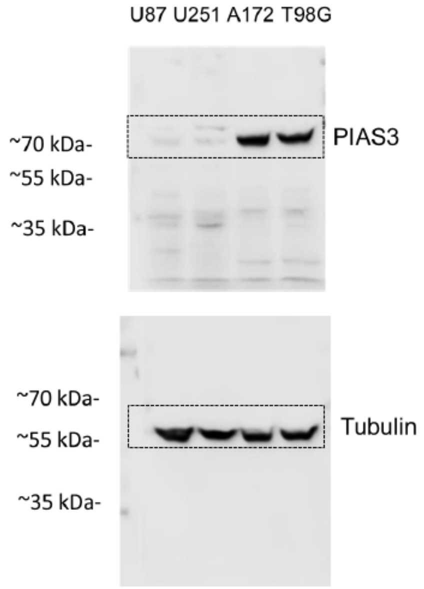

S Fig. 6a

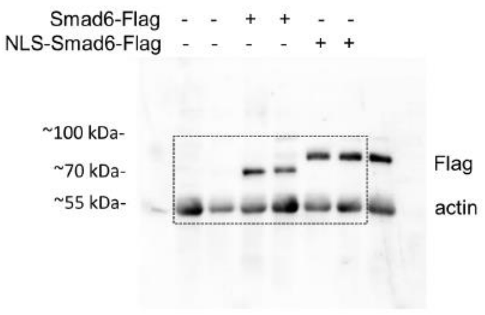

S Fig. 6c

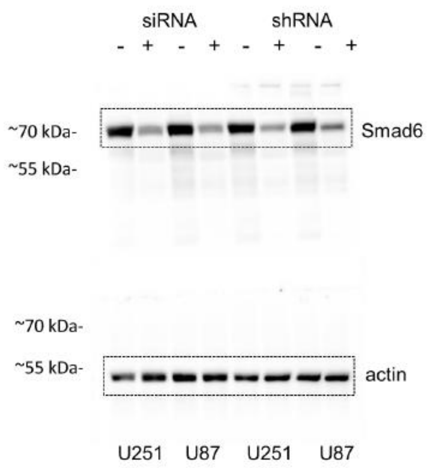

S Fig. 8a

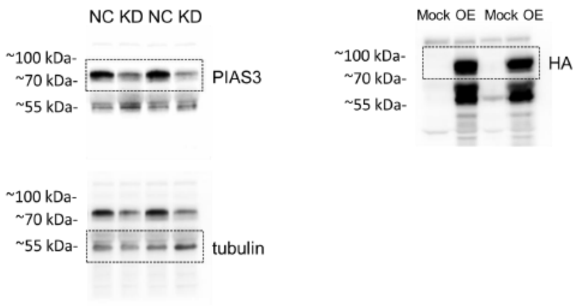

S Fig. 11

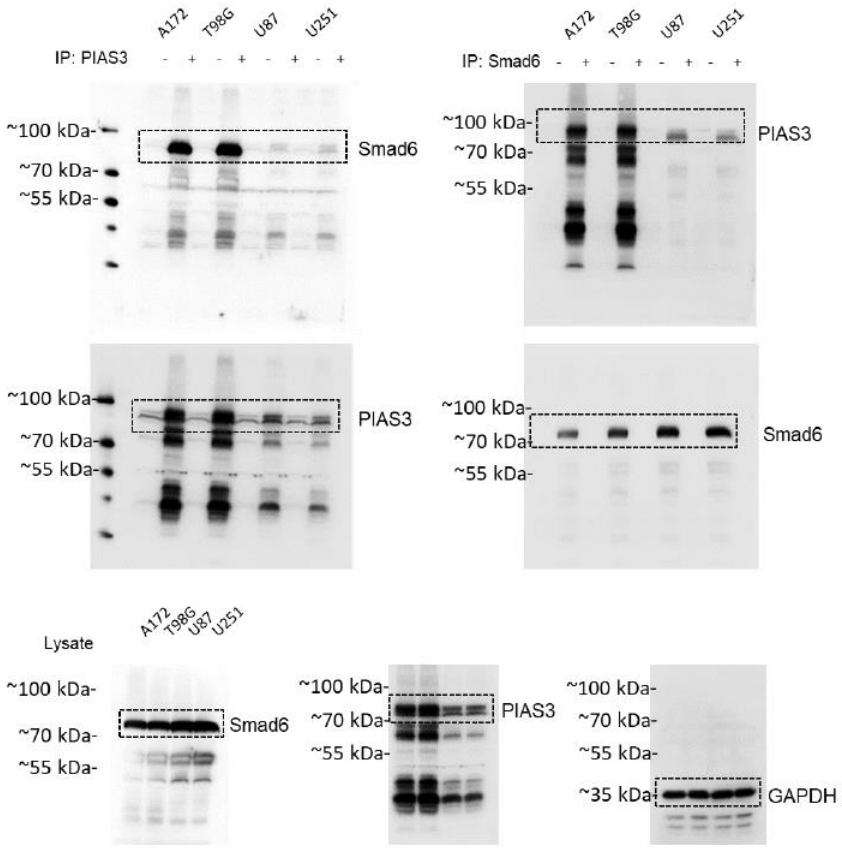

S Fig. 13 (left)

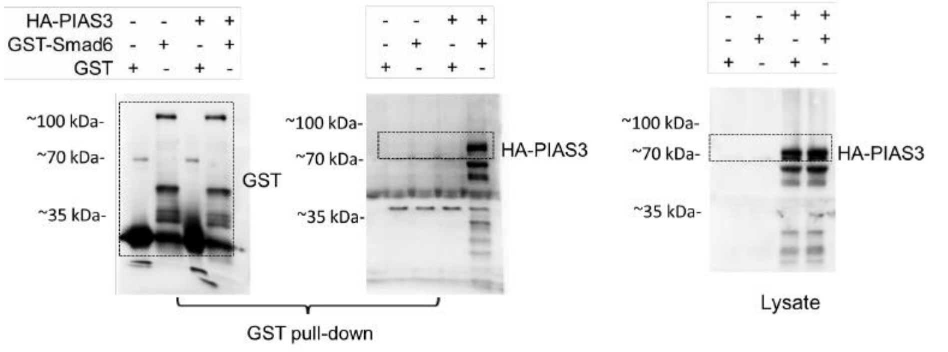

S Fig. 13 (right)

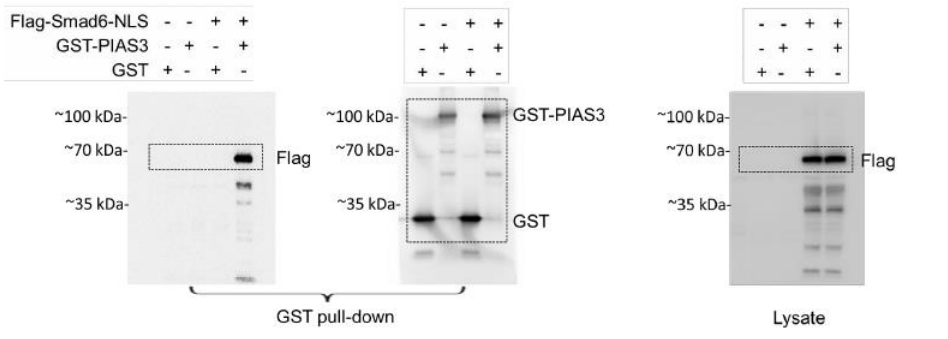

S Fig. 14b

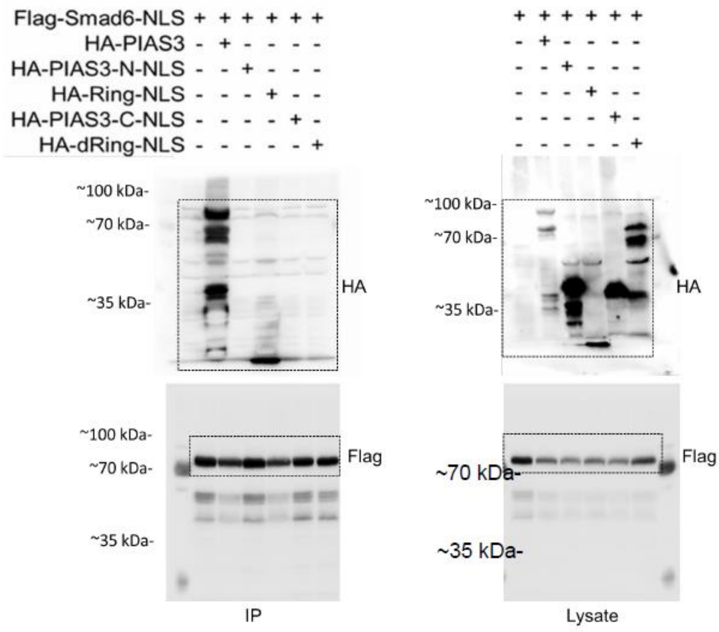

S Fig. 14c

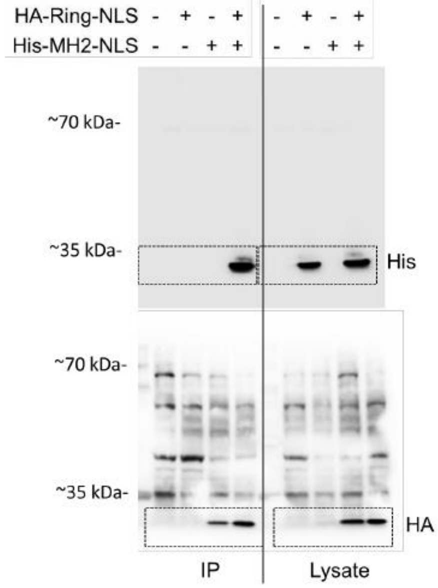

S Fig. 15a

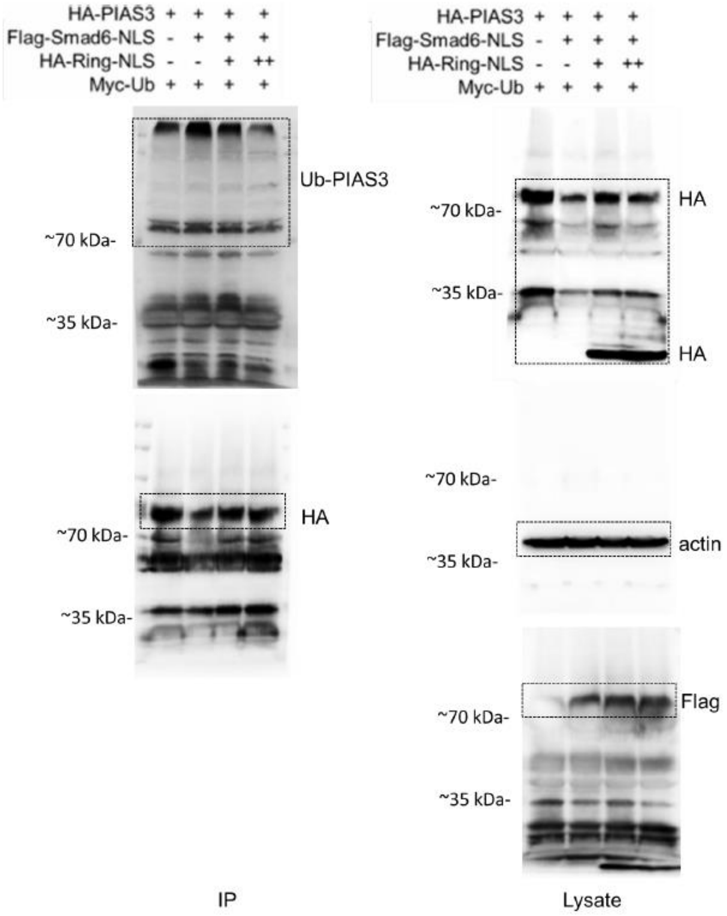

S Fig. 15b

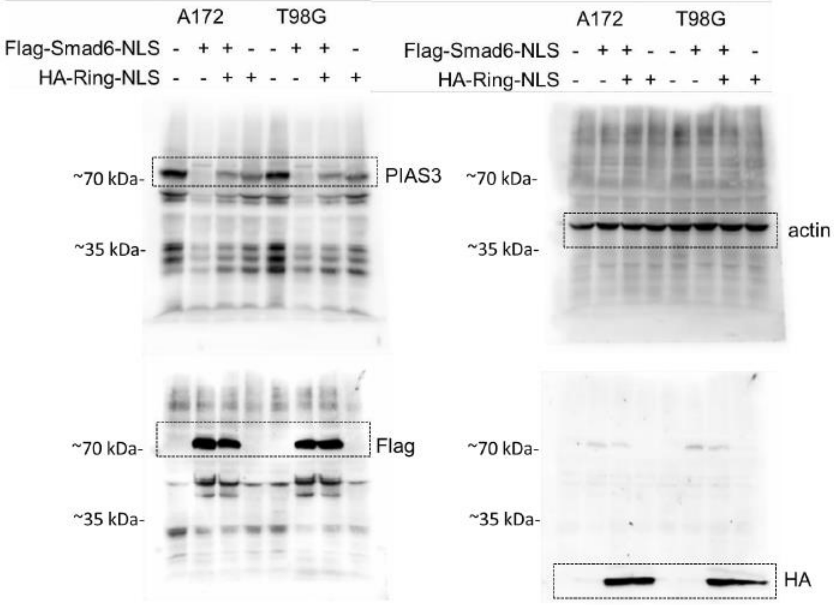

S Fig. 15c

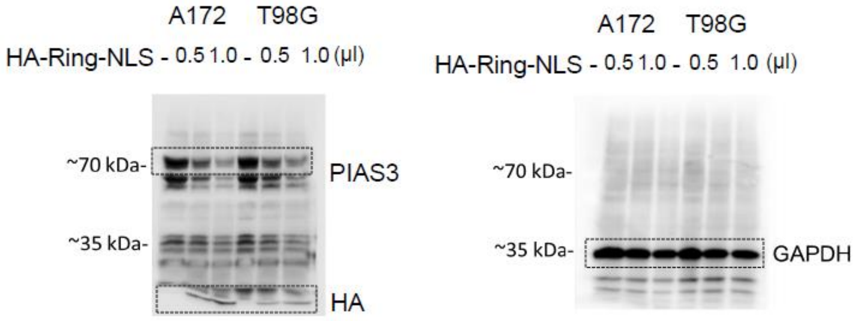

S Fig. 15d

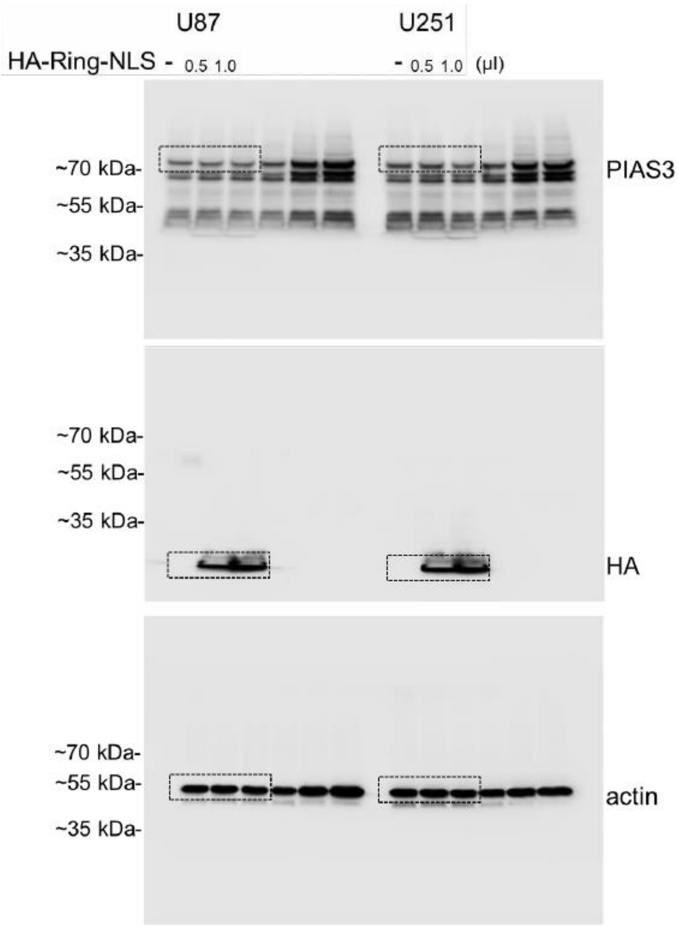

Supplementary Figure 17. The uncropped blots.

Supplementary Table 1. Correlation between Smad6 expression and information of patients with glioma

Status		Smad6 expression#, n		Total	*P* value*
		LOW	HIGH	n	
Gender	Male	33	43	76	0.905
	Female	28	38	66	
Age	<55 yrs	23	38	57	0.607
	≥55 yrs	34	47	85	
Survival$	≤1 yrs	12	13	25	0.193
	1-3 yrs	12	24	36	
	>3 yrs	19	16	35	
Grade	II	10	8	18	0.134
	III	13	29	42	
	IV	38	44	82	

**P* values were analyzed by Chi-square test.
According to the immunoreactive scores (IRS) from IHC of glioma tissue array: the cutoff between LOW and HIGH was set at the mean IRS of normal brains.
$ The total number of patients used in survival analysis was 96, those without survival information were excluded.

Supplementary Table 2. Correlation between PIAS3 expression and information of patients with glioma

Status		PIAS3 expression#, n		Total	*P* value*
		LOW	HIGH	n	
Gender	Male	65	11	76	0.407
	Female	53	13	66	
Age	<55 yrs	50	7	57	0.229
	≥55 yrs	68	17	85	
Survival	≤1 yrs	23	2	25	0.122
	1-3 yrs	30	6	36	
	>3 yrs	25	10	35	
Grade	II	16	2	18	0.752
	III	34	8	42	
	IV	68	14	82	

**P* values were analyzed by Chi-square test
The cutoff between LOW and HIGH was set at the mean IRS of normal brains.
$ The total number of patients used in survival analysis was 96, those without survival information were excluded.

Supplementary Table 3. Patient's information in TCGA GBM dataset

TCGA GBM patient's information	
**Characteristics**	**GBM (n=327)**
**Gender**	
Male	206
Female	121
**Age at diagnoss**	
Mean $\pm$ SD	56.3$\pm$14.3
Median (Range)	57.5(14.5-86.6)
**Subtype**	
Classical	88
Mesenchymal	99
Neural	53
Proneural	76
Unclassified	11

Supplementary Table 4. List of antibodies

Antigen	Primary Antibody	Dilution
Smad6	Sigma;SAB4200383:;rabbit polyclonal	1:2,000 for IHC/IF;1:1,000 for WB; 1:100 for IP
Smad6	ABCAM; ab13727; rabbit polyclonal	1:1000 for WB
PIAS3	Cell Signaling; 9042; rabbit monoclonal	1:500 for IHC/IF; 1:1000 for WB; 1:100 for IP
PCNA	ABCAM; ab92552; rabbit monoclonal	1:2000 for WB
c-jun	Cell Signaling; 9165; rabbit monoclonal	1:1000 for WB
CCND1	Cell Signaling; 2978; rabbit monoclonal	1:1000 for WB
Sox2	Epitomics; 2683-1; rabbit monoclonal	1:2000 for WB
STAT3	Cell Signaling;9139; mouse monoclonal	1:1000 for WB; 1:100 for ChIP
Phospho-STAT3 (Tyr705)	Cell Signaling; 9145; rabbit monoclonal	1:1000 for WB
Histone H3	Cell Signaling; 4620; rabbit monoclonal	1:50 for ChIP
Normal Rabbit IgG	Cell Signaling; 2729	1:100 for IP or ChIP
β-actin	GeneTex; GTX109639; rabbit polyclonal	1:1000 for WB
GAPDH	GeneTex; GTX1001118;	1:5000 for WB

	rabbit polyclonal	
β-tubulin	Sigma;T5201; mouse monoclonal	1:200 for IF
β-tubulin	Santa Cruz; sc-9104; rabbit polyclonal	1:1000 for WB
Tuj1	ABCAM; ab78078; mouse monoclonal	1:800 for IF
Ki67	ABCAM; ab16667; rabbit monoclonal	1:400 for IF
Flag-tag	Sigma; F7425; rabbit polyclonal	1:400 for IF; 1:2000 for WB; 1:200 for IP
GFP-tag	Cell Signaling; 2956; rabbit monoclonal	1:2000 for WB
HA-tag	Cell Signaling; 3724; rabbit monoclonal	1:2000 for WB; 1:50 for IP
HA-tag	Abmart; M20003; mouse monoclonal	1:1000 for IF
His-tag	Abmart; M30111; mouse monoclonal	1:1000 for IF; 1:5000 for WB; 1:100 for IP
Myc-tag	Cell Signaling; 2276; mouse monoclonal	1:2000 for WB; 1:500 for IP
GST	ABCAM; ab19256; rabbit monoclonal	1:2000 for WB
Ubiquitin	ABCAM; ab7780; rabbit monoclonal	1:1000 for WB; 1:100 for IP

Supplementary Table 5. Primers used for qPCR

gene	forward	reverse
SMAD6	CACTGAAACGGAGGCTACCAAC	CCTGGTCGTACACCGCATAGAG
PIAS3	ACTCTCAGCCACTGTTCCCAAC	CAGTCAACTGCCTCACCAGGTA
GAPDH	CAACTTTGGTATCGTGGAAGGACTC	AGGGATGATGTTCTGGAGAGCC
