# Supplementary material for: Nuclear Smad6 promotes gliomagenesis by negatively regulating PIAS3-mediated STAT3 inhibition
Source: Nat Commun. 2018 Jun 27;9:2504. doi: 10.1038/s41467-018-04936-9 (PMC6021382; doi:10.1038/s41467-018-04936-9)
Supplement: Supplementary file 2 — Description of Additional Supplementary Files [file 41467_2018_4936_MOESM2_ESM.pdf]

Description of Additional Supplementary Files

File Name: Supplementary Data 1
Description: Clinical pathological information of Gliomas.

File Name: Supplementary Data 2
Description: SMAD6 and PIAS3 expression in TCGA GBM dataset.
